# Association of regular glucosamine use with incident dementia: evidence from a longitudinal cohort and Mendelian randomization study

**DOI:** 10.1186/s12916-023-02816-8

**Published:** 2023-03-29

**Authors:** Jiazhen Zheng, Can Ni, Yingchai Zhang, Jinghan Huang, Daniel Nyarko Hukportie, Buwen Liang, Shaojun Tang

**Affiliations:** 1 Bioscience and Biomedical Engineering Thrust, Systems Hub, The Hong Kong University of Science and Technology (Guangzhou), Guangzhou, Guangdong China; 2 Department of Medicine and Therapeutics, Prince of Wales Hospital, The Chinese University of Hong Kong, Sha Tin, New Territories, Hong Kong, SAR China; 3 Biomedical Genetics Section, School of Medicine, Boston University, Boston, USA; 4 Department of Chemical Pathology, Faculty of Medicine, The Chinese University of Hong Kong, Hong Kong, SAR China; 5 Department of Epidemiology, School of Public Health, (Guangdong Provincial Key Laboratory of Tropical Disease Research), Southern Medical University, Guangzhou, China; 6 Division of Emerging Interdisciplinary Areas, The Hong Kong University of Science and Technology, Clear Water Bay, Hong Kong, SAR China

**Keywords:** Glucosamine, Dementia, Alzheimer’s disease, APOE

## Abstract

**Background:**

Emerging data suggests the neuroprotective and anti-neuroinflammatory effects of glucosamine. We aimed to examine the association between regular glucosamine use and risk of incident dementia, including dementia subtypes.

**Methods:**

We conducted large-scale observational and two-sample Mendelian randomization (MR) analyses. Participants in UK Biobank having accessible data for dementia incidence and who did not have dementia at baseline were included in the prospective cohort. Through the Cox proportional hazard model, we examined the risks of incident all-cause dementia, Alzheimer’s disease (AD), and vascular dementia among glucosamine users and non-users. To further test the causal association between glucosamine use and dementia, we conducted a 2-sample MR utilizing summary statistics from genome-wide association studies (GWAS). The GWAS data were obtained from observational cohort participants of mostly European ancestry.

**Results:**

During a median follow-up of 8.9 years, there were 2458 cases of all-cause dementia, 924 cases of AD, and 491 cases of vascular dementia. In multivariable analysis, the hazard ratios (HR) of glucosamine users for all-cause dementia, AD, and vascular dementia were 0.84 (95% CI 0.75–0.93), 0.83 (95% CI 0.71–0.98), and 0.74 (95% CI 0.58–0.95), respectively. The inverse associations between glucosamine use and AD appeared to be stronger among participants aged below 60 years than those aged above 60 years (*p* = 0.04 for interaction). The *APOE* genotype did not modify this association (*p* > 0.05 for interaction). Single-variable MR suggested a causal relationship between glucosamine use and lower dementia risk. Multivariable MR showed that taking glucosamine continued to protect against dementia after controlling for vitamin, chondroitin supplement use and osteoarthritis (all-cause dementia HR 0.88, 95% CI 0.81–0.95; AD HR 0.78, 95% CI 0.72–0.85; vascular dementia HR 0.73, 95% CI 0.57–0.94). Single and multivariable inverse variance weighted (MV-IVW) and MR-Egger sensitivity analyses produced similar results for these estimations.

**Conclusions:**

The findings of this large-scale cohort and MR analysis provide evidence for potential causal associations between the glucosamine use and lower risk for dementia. These findings require further validation through randomized controlled trials.

**Supplementary Information:**

The online version contains supplementary material available at 10.1186/s12916-023-02816-8.

## Background

Dementia is characterized by an inexorably progressive impairment of cognition and the capacity to carry out activities of daily life. It is a heterogeneous syndrome posing a substantial burden on patients, their proxies, and national health-care systems [1]. In the UK, over 850,000 individuals suffer with dementia [2]. Globally, roughly 50 million individuals have dementia, with this figure expected to rise to 152 million by 2050 [1]. In the absence of effective pharmacological treatments for dementia, the identification and detailed investigation of potentially modifiable protective factors have gained considerable attention in recent years.

Glucosamine is a widely used non-vitamin, non-mineral supplement for relieving both osteoarthritis and joint discomfort [3]. It is an approved osteoarthritis prescription medication in most European nations and is widely used as a nutritional supplement in countries like the USA and Australia, where roughly 20% of adults use it daily [4, 5]. Despite the controversy regarding the efficacy of glucosamine supplements on osteoarthritis and joint discomfort [6, 7], glucosamine has been proved to have anti-inflammatory properties [8] and may prevent a wide range of diseases [9, 10]. In this instance, a variety of epidemiological studies have revealed that glucosamine consumption may protect against colorectal cancer [11, 12], lung cancer, [13], cardiovascular disease [14, 15], diabetes [16], and all-cause death [17]. Importantly, a cross-sectional research recorded the association between glucosamine consumption and better cognitive function [18]. However, research regarding the association between glucosamine use and dementia risk remains scant.

The importance of glucosamine in brain function has been highly supported by previous studies [19, 20]. Glucosamine mimicked the effects of a low-carbohydrate diet in a prior animal research, resulting in increased lifespan [21], and studies consistently showed that a low-carbohydrate diet protects against dementia [22, 23]. An animal study suggested that glucosamine may promote cognitive function by impacting energy metabolism [20]; other animal models have indicated the neuroprotective and anti-neuroinflammatory effects of glucosamine [24]. In addition, glucosamine participates in the O-linked N-acetylglucosaminylation of various proteins, which was verified to be related to many neurological or neurodegenerative diseases [25, 26]. Therefore, we hypothesize that regular use of glucosamine may have a causal influence on incident dementia.

Based on the UK Biobank study of nearly 500,000 British people, we investigated the relationship between regular use of glucosamine and the risk of all-cause dementia, Alzheimer’s disease (AD) and vascular dementia. We also explored potential modifying effects by several established risk factors (including *APOE* ε4 genotypes) for dementia.

Traditional observational studies include drawbacks such as residual confounding and/or reverse causation, inadequate adjustment (e.g., healthy lifestyle or other factors), and a focus on correlation rather than causation. By employing genetic variants as proxy for glucosamine use, Mendelian randomization (MR) avoids some of these limitations and provides genetic support for causal associations [27]. Thus, in addition to observational analysis, we performed MR to give additional insights for the assessment of potential causal relationships.

## Methods

### Study design

**Fig. 1:**
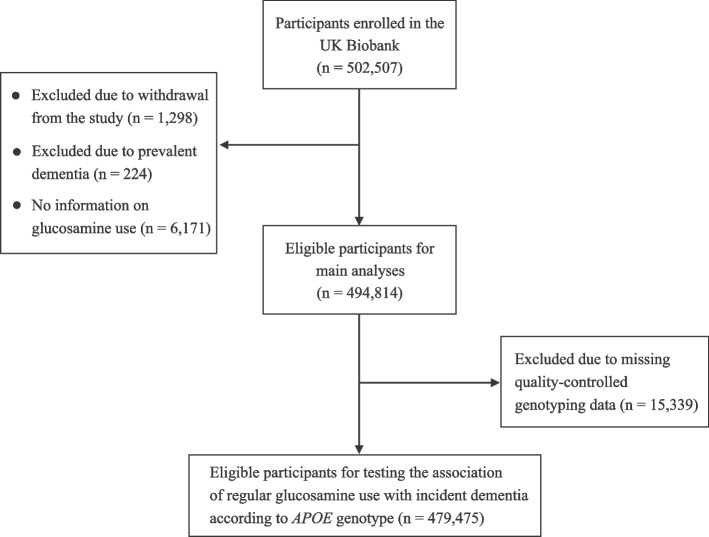
Flow diagram of the participant selection process

This study analyzed data from UK Biobank (application 55,794), a large prospective cohort study enrolling over 500,000 participants between the ages of 40 and 70 from 22 research centers in the UK (England, Wales, and Scotland) between 2006 and 2010 [28]. Through detailed electronic questionnaires, face-to-face interviews, and physical assessments, participants provided personal data on health-related variables. Participants who dropped out of the study (*n* = 1298), had dementia (*n* = 224), or lacked data on glucosamine use (*n* = 6171) were excluded from the analysis. We also excluded 15,339 participants from further analysis owing to a lack of quality-controlled genotyping data (Fig. 1).

### Exposure assessment

At one of 22 assessment sites across the UK, the participants filled out a touch-screen questionnaire. In answer to the question “Do you usually take any of the following?”, a list of supplements, which included glucosamine, was given to the participants to choose from. Based on these data, we established a binary classification for regular glucosamine use: 1 = yes, 0 = no. This evaluation method was used in previous studies [16, 29, 30].

### Ascertainment of incident dementia

We used participants’ baseline survey information, hospital admission diagnosis records, and death registration records to define outcomes, which included all-cause dementia, AD, and vascular dementia. Diagnoses were recorded using the International Classification of Diseases (ICD) coding system (Additional file 1: Table S1) [31]. The incident disease in this study was determined by the primary or secondary diagnoses from hospital admission data or primary or secondary causes of inducing death after baseline data collection. A subsample of the population was also retrieved from primary care data using Read Codes (version 2 or 3) in the sensitivity analysis [32]. Participants were followed up from the time of the baseline to the first diagnosis, death, or February 25, 2018, in Wales and England and February 28, 2017, in Scotland, whichever came first. Detailed information on the *APOE* genotyping is presented in the Additional file 1: Supplemental Methods [33–35].

### Covariates

Various potential confounders were assessed using a baseline touch-screen questionnaire. Age, gender, ethnicity, the Townsend Deprivation Index (TDI), level of education, and annual household income were included as the sociodemographic factors. Lifestyle behavior included smoking status, alcohol intake, being physically active, body mass index (BMI), vegetable intake and fruit intake. Health-related variables included hypertension, cardiovascular disease, cancer, digestive disease, depression, diabetes, emphysema or chronic bronchitis, high cholesterol, chronic kidney disease, chronic liver disease, and Elixhauser Comorbidity Index. Medication utilization included antihypertensive drugs, insulin treatment, statin, opioids, aspirin, and other non-steroidal anti-inflammatory drugs (NSAIDs). We also included chondroitin, dietary supplements for minerals, vitamins, and other nutrients (fish oil, calcium, iron, zinc, and selenium), memory, and reaction time in the analysis. The details on calculating the Elixhauser Comorbidity Index are shown in the Additional file 1: Supplemental Methods [36, 37]. We calculated participants’ BMI by dividing their weight by the square of their height in meters. The TDI is a comprehensive poverty index, which is calculated by the following factors: ownership of a home, ownership of a car, being unemployed or not, and whether there are too many people living together [38]. It shows the socioeconomic status of a participant. According to WHO guidelines on physical activity for health [39], we classified individuals as < 150 or ≥ 150 min/week based on total minutes of moderate physical activity per week (collected by touchscreen question, one vigorous physical activity minute equals two moderate physical activity minutes). This assessment method was widely used in prior studies [40, 41]. Patients with any of the following situations are classified as having hypertension: using hypertensive drugs, systolic blood pressure higher than 140 mmHg, diastolic blood pressure higher than 90 mmHg, or self-reported hypertension. Health status was determined through self-reporting combined with ICD-10 codes from hospital records. Memory and reaction time assessments were conducted through touch screen [42–44]. A pair matching test was used to measure memory, in which participants had to recall six pairs of shapes and their positions in 5 s. The number of mistakes made during matching was used to evaluate performance. The test method of reaction time is shown below: a series of figures will be displayed on the screen; the participant was asked to press the button as rapidly as possible when two identical figures appear. The mean response time (ms) across eight rounds for properly selected matching groups was used to measure performance. The UK Biobank website has further information on these variables (www.ukbiobank.ac.uk).

### Statistical analysis

#### Observational analysis

For continuous variables, the mean (SD) is used, and for categorical variables, the number (%) is used. We performed multiple imputation with chained equations to cope with missing variables to reduce the possibility of inferential bias [45, 46]. There were five datasets imputed. The imputation model contained all variables used in the analysis. Additional file 1: Table S2 provides detailed data on missing variables.

Cox proportional hazard models were used to calculate the hazard ratios (HR) and 95% confidence intervals (CIs) for the relationships between regular glucosamine use and all-cause dementia, AD, and vascular dementia. We tested the proportional hazards assumption by Schoenfeld residual tests [47], and no violations of this assumption were identified. Two models were used. We only included sex and age in Model 1. Additional factors, such as ethnicity, education, TDI, annual household income, BMI, fruit intake, vegetable intake, smoking status, alcohol intake, being physically active, medical conditions, drug use, other supplement use, memory, and reaction time were adjusted in Model 2.

In order to evaluate potential effect modifiers, subgroup analyses based on sex (female or male), age (< 60 or ≥ 60 years), obesity (BMI ≥ 30 kg/m^2^, no or yes), current smoking status (no or yes), diabetes (no or yes), hypertension (no or yes), aspirin use (no or yes), use of non-aspirin NSAIDs (no or yes), use of vitamin supplementation (no or yes), use of other non-vitamin supplementation (no or yes) and *APOE* ε4 carrier (no or yes) were performed. To investigate the differential effects of glucosamine on the likelihood of dementia in subgroups, we calculated the p-value for interaction by including the cross-product term of the stratifying variables with glucosamine use in the fully adjusted model.

We evaluated the robustness of our findings by a sequence of sensitivity analyses (Additional file 1: Table S3). Firstly, we conducted an analysis of competing risks which considered all-cause mortality as a competing event for dementia. Secondly, since individuals who took glucosamine were more likely to take chondroitin than those who did not, we conducted sensitivity analyses by removing chondroitin users. Thirdly, to minimize the possibility of reverse causality, we excluded people who died within the first 2 years after baseline assessment. Fourthly, we excluded individuals who had missing covariate values. Fifthly, we calculated a propensity score for each participant and further adjusted for the score in the fully adjusted model. The multivariate logistic regression model was applied to estimate propensity scores taking all covariates into account. Sixthly, we added a subsample of the population retrieved from primary care data using Read Codes (version 2 or 3) in the analysis. We performed all analyses using R version 4.0.3, and *p* less than 0.05 (two-sided) was deemed significant.

#### Mendelian randomization

We performed a two-sample MR design using summary-level data. Single-nucleotide polymorphisms (SNPs) served as risk factor instruments. The analysis relied on public summary-level data. All original studies received ethical approval. A detailed description of the data sources, the selection of genetic instrumental variables, and the test on instrument strength and statistical power are shown in the Additional file 1: Supplemental Methods [48–56]. Data sources and instruments are listed in Additional file 1: Table S4-12. The GWAS from Ben Neale Lab round 2 used a linear regression model in Hail for large-scale phenotypes in the UK Biobank, even for binary variables. As a workaround, we used BOLT-LMM, a software package widely used to deal UK Biobank data, to calibrate the effect. The detailed information on BOLT-LMM is shown in the Additional file 1: Supplemental Methods [57].

The “MendelianRandomization” and “TwoSampleMR” R packages were used for all statistical analyses. Inverse variance-weighted (IVW) MR was the major analysis we applied for single-variable MR analysis. In order to deal with the issue of the robustness of the IVW result, we used the MR-Egger and weighted median-based regression, both of which assume distinct instrumental variables assumptions [54, 58]. When all genetic variants are invalid instrumental variables, the MR-Egger regression produces consistent results; the weighted median needs valid IVs to contribute 50% of the weight. The accuracy of weighted median estimates and IVW estimates are almost the same, which are much higher than that of MR-Egger estimates because the accuracy of MR-Egger estimates is particularly imprecise when all the IVs are about the same strength [59]. To assess potential IV violations, we carried out the MR-Egger intercept test [60], MR pleiotropy residual sum and outlier (MR-PRESSO) test [61], and Cochran Q heterogeneity test [62]. To identify high-influence points, we used a leave-one-out validation [63]. The MR Steiger test was also performed to assess the potential reverse causal effect of glucosamine on dementia (Additional file 1: Table S13) [64].

The likelihood of using additional supplements is higher among glucosamine users than in non-glucosamine users. Taking these associations into account, we conducted multivariable MR to assess the direct effect of regular use of glucosamine on dementia under the condition of controlling vitamin, chondroitin supplements intake, and osteoarthritis. We aggregated the genetic instruments used in the related GWASs—glucosamine, vitamin, chondroitin supplements, and osteoarthritis. SNPs were clumped by linkage disequilibrium within a window of 10,000 kb (*R*^2^ < 0.001) to confirm their independence. Then we derived SNP effects and standard errors from the GWAS summary statistics and harmonized them with GWAS data on dementia. Measured and unmeasured pleiotropy were taken into account by using multivariable MR extension of the IVW MR approach [65] and the MR-Egger method [66].

## Results

The mean age of the 494,814 participants was 56.5 years (SD 8.1) and the proportion of female was 54.4%. At baseline, 94,259 (19.0%) of participants reported using glucosamine. There was a higher percentage of older, female, non-smoking, and physically active glucosamine users than nonusers. In addition, glucosamine users had a lower TDI, higher prevalence of comorbidities such as cancer, hypertension, arthritis, and depression, but less cardiovascular disease, emphysema or chronic bronchitis, diabetes, high cholesterol, chronic kidney disease, and chronic liver disease. The percentage of using statin, opioids, non-aspirin NSAIDs, chondroitin, vitamins, minerals, and other dietary supplements was higher in glucosamine users than non-users (Table 1).

**Table 1:** Baseline characteristics of study participants by glucosamine use

	**All participants** **(*n* = 494,814)**	**Use of glucosamine**	
		**Yes (*n* = 94,259)**	**No (*n* = 400,555)**
**Age, mean (SD), years**	56.54 (8.09)	59.08 (7.07)	55.95 (8.20)
**Female**	269,380 (54.4)	58,996 (62.6)	210,384 (52.5)
**White ethnicity**	466,252 (94.2)	90,306 (95.8)	375,946 (93.9)
**With college or university degree**	160,409 (32.4)	31,119 (33.0)	129,290 (32.3)
**TDI, mean (SD)**	-1.31 (3.09)	-1.79 (2.79)	-1.20 (3.14)
**Household income (£)**			
18,000	116,776 (23.6)	21,044 (22.3)	95,732 (23.9)
≥ 18,000	378,038 (76.4)	73,215 (77.7)	304,823 (76.1)
**BMI, mean (SD), kg/m**^**2**^	27.43 (4.80)	27.36 (4.65)	27.45 (4.83)
**Physical activity (min/week)**			
150	228,109 (46.1)	38,269 (40.6)	189,840 (47.4)
≥ 150	266,705 (53.9)	55,990 (59.4)	210,715 (52.6)
**Fruit intake (servings/day)**			
4	337,958 (68.3)	56,085 (59.5)	281,873 (70.4)
≥ 4	156,856 (31.7)	38,174 (40.5)	118,682 (29.6)
**Vegetable intake(servings/day)**			
4	320,640 (34.8)	57,027 (60.5)	263,613 (65.8)
≥ 4	174,174 (35.2)	37,232 (39.5)	136,942 (34.2)
**Alcohol consumption frequency**			
3 times a week	280,147 (56.6)	49,736 (52.8)	230,411 (57.5)
≥ 3 times a week	214,667 (43.4)	44,523 (47.2)	170,144 (42.5)
**Smoking status**			
Never smoker	271,869 (54.9)	52,132 (55.3)	219,737 (54.9)
Ex-smoker	170,903 (34.5)	36,013 (38.2)	134,890 (33.7)
Current smoker	52,042 (10.5)	6114 (6.5)	45,928 (11.5)
**Personal medical condition**			
Hypertension	279,569 (56.5)	54,670 (58.0)	224,899 (56.1)
CVD	28,699 (5.8)	4147 (4.4)	24,552 (6.1)
Cancer	39,090 (7.9)	7823 (8.3)	31,267 (7.8)
Arthritis	23,256 (4.7)	7729 (8.2)	15,527 (3.9)
Emphysema or chronic bronchitis	8262 (1.7)	1325 (1.4)	6937 (1.7)
Diabetes	25,945 (5.2)	3450 (3.7)	22,495 (5.6)
High cholesterol	86,314 (17.4)	15,972 (16.9)	70,342 (17.6)
Digestive disease	1484 (0.3)	188 (0.2)	1296 (0.3)
Chronic kidney disease	10,391 (2.1)	1885 (2.0)	8506 (2.1)
Chronic liver disease	7422 (1.5)	1225 (1.3)	6197 (1.5)
Depression	75,706 (15.3)	14,704 (15.6)	61,002 (15.2)
Elixhauser Comorbidity Index, mean (SD)	2.1 (1.7)	2.3 (1.8)	2.0 (1.7)
**Medication or supplementation**			
Antihypertensive drugs	88,208 (17.8)	16,897 (17.9)	71,311 (17.8)
Insulin treatment	4839 (1.0)	594 (0.6)	4245 (1.1)
Use of statin	55,913 (11.3)	10,839 (11.5)	45,074 (11.3)
Use of opioids	26,719 (5.4)	5372 (5.7)	21,347 (5.3)
Use of aspirin	69,216 (14.0)	13,299 (14.1)	55,917 (14.0)
Use of non-aspirin NSAIDs	72,939 (14.7)	17,713 (18.8)	55,226 (13.8)
Use of chondroitin	6432 (1.3)	5844 (6.2)	588 (0.1)
Use of vitamin supplementation	157,109 (31.8)	52,388 (55.6)	104,721 (26.1)
Use of minerals and other dietary supplementation	184,233 (37.2)	65,352 (69.3)	118,881 (29.7)
**Memory, mean (SD), no. of errors**	4.25 (3.32)	4.28 (3.45)	4.23 (3.34)
**Reaction time, mean (SD), ms**	558 (118)	562 (125)	557 (120)
APOE*E4 carrier	135,883 (28.3)	25,609 (28.0)	110,274 (28.4)

Values are numbers (%) unless stated otherwise. *TDI*, Townsend Deprivation Index; *BMI*, body mass index; *CVD*, cardiovascular disease; *NSAID*, non-steroidal anti-inflammatory drug; *APOE*, apolipoprotein E. All variables globally significantly different between groups at *P* < 0.001, except for BMI, digestive disease and use of aspirin (*P* > 0.05). *P*-values are derived using either Student’s t-test, Wilcoxon rank sum test, or chi-square test

### Associations of glucosamine use with incident dementia

During the 8.9-year (IQR 8.3–9.7 years) median follow-up, we recorded 2458 cases of all-cause dementia, 924 cases of AD, and 491 cases of vascular dementia. Table 2 shows the associations of regular use of glucosamine with the outcomes. A statistically significant inverse relationship was found between glucosamine use and risk for all-cause dementia (HR 0.81; 95% CI 0.73–0.90), AD (HR 0.78; 95% CI 0.65–0.92), and vascular dementia (HR 0.68; 95% CI 0.54–0.87). The hazard ratios of glucosamine users in multivariable-adjusted models were 0.84 (95% CI 0.75 to 0.93) for all-cause dementia; 0.83 (95% CI 0.71 to 0.98) for AD; and 0.74 (95% CI 0.58 to 0.95) for vascular dementia (Table 2).

**Table 2:** Associations of regular glucosamine use with incident dementia

Outcomes	Glucosamine non-user **(*n* = 400,555)**	Glucosamine user **(*n* = 94,259)**	Model 1^a^		Model 2^b^		Propensity score adjusted	
			**HR (95%CI)**	***P* value**	**HR (95%CI)**	***P* value**	**HR (95%CI)**	***P* value**
All-cause dementia	1971 (0.5)	487 (0.5)	0.81 (0.73–0.90)	< 0.001	0.84 (0.75–0.93)	0.002	0.82 (0.73–0.92)	< 0.001
Alzheimer’s disease	732 (0.2)	192 (0.2)	0.78 (0.65–0.92)	0.018	0.83 (0.71–0.98)	0.029	0.80 (0.68–0.95)	< 0.001
Vascular dementia	408 (0.1)	83 (0.09)	0.68 (0.54–0.87)	0.002	0.74 (0.58–0.95)	0.018	0.72 (0.56–0.93)	0.009

Values are numbers (%) unless stated otherwise

^a^Model 1: adjusted for age and sex

^b^Model 2: additionally adjusted for ethnicity, education, Townsend Deprivation Index, household income, body mass index, fruit consumption, vegetable consumption, smoking status, alcohol consumption, physical activity, health condition, antihypertensive drugs, insulin treatment, statin use, opioids use, chondroitin use, aspirin use, non-aspirin NSAID use, vitamin supplementation, mineral and other dietary supplementation, memory, and reaction time

### Subgroup and sensitivity analyses

**Fig. 2:**
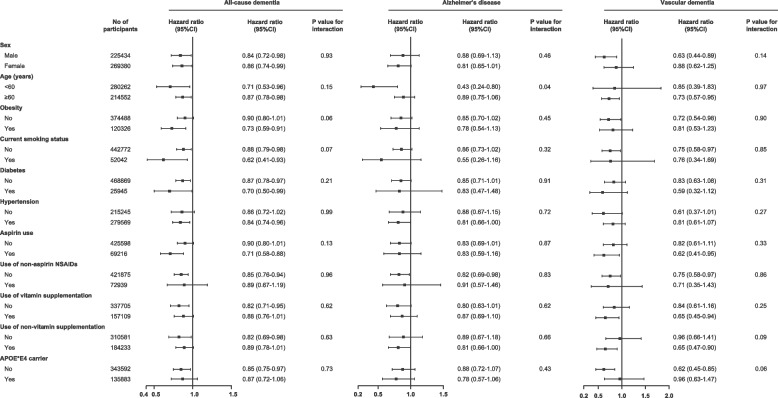
Relationship between glucosamine use and risk of dementia stratified by potential risk factors. Findings were adjusted for age, sex, ethnicity, education, Townsend Deprivation Index, household income, body mass index, fruit consumption, vegetable consumption, smoking status, alcohol consumption, physical activity, health condition, antihypertensive drugs, insulin treatment, statin use, opioids use, chondroitin use, aspirin use, non-aspirin NSAID use, vitamin supplementation, mineral and other dietary supplementation, memory, and reaction time

To investigate potential subgroup effects, we conducted several specified subgroup analyses (Fig. 2). We found that the protective effect of glucosamine on AD was stronger among participants aged below 60 years, compared with those above 60 years (*p* = 0.04 for interaction). Other stratifying variables have not modified the association of glucosamine use with incident dementia (*p* for interaction > 0.05).

When we excluded participants who had outcomes within 2 years of follow-up, participants who used chondroitin, and participants with missing values for variables, the relationships of glucosamine use with all-cause dementia, AD, and vascular dementia persisted. After adding cases that were retrieved from the primary care data using Read Codes, the results did not alter. During the follow-up in participants without dementia, 19,082, 19,654, and 19,763 deaths were documented as competing events for all-cause dementia, AD, and vascular dementia, respectively. The competing risks analysis produced results which were consistent with the Cox proportional hazards model (Additional file 1: Table S2).

### Mendelian randomization

According to Univariable MR analysis, genetically determined regular glucosamine use was associated with a decreased risk for all-cause dementia (IVW odds ratio, 0.85; 95% CI 0.76 to 0.95), AD (IVW odds ratio, 0.85; 95% CI 0.78 to 0.93) and vascular dementia (IVW odds ratio, 0.64; 95% CI 0.42 to 0.96) (Table 3). Weighted median and the MR-Egger provided similar estimates to those of IVW. The accuracy of the MR-Egger estimations was much lower. We use forest plots to display the MR results for the impacts of SNPs related to glucosamine use on dementia risk (Additional file 1: Fig. S1). No pleiotropy across instruments has been found by the Cochran’s Q statistic. We did not find directional pleiotropy by MR-Egger intercept analysis. No potential outliers were found by MR-PRESSO. No high leverage, high impact points were found using conventional IVW leave-one-out analysis (Additional file 1: Fig. S2). Absence of weak instrument bias is shown by F statistics for genetic instruments (Additional file 1: Table S5). Using the MR Steiger test, we detected no evidence of reverse causality (Additional file 1: Table S13).

**Table 3:** MR results for the relationship between regular glucosamine use and incident dementia

**Method**	**All-cause dementia**				**Alzheimer’s disease**				**Vascular dementia**			
	**Number of SNPs**	**OR (95% CI)**	***P* for association**	***P* for MR-Egger** **intercept**	**Number of SNPs**	**OR (95% CI)**	***P* for association**	***P* for MR-Egger** **intercept**	**Number of SNPs**	**OR (95% CI)**	***P* for association**	***P* for MR-Egger** **intercept**
**Univariable MR**												
IVW	9	0.85 (0.76–0.95)	0.007	0.542	9	0.85 (0.78–0.93)	< 0.001	0.505	9	0.64 (0.42–0.96)	0.031	0.763
Weighted median		0.83 (0.72–0.96)	0.011			0.85 (0.76–0.96)	0.007			0.44 (0.30–0.65)	< 0.001	
MR-Egger		0.32 (0.03–3.49)	0.378			0.50 (0.08–3.07)	0.481			0.82 (0.05–6.15)	0.762	
**Multivariable MR**^**a**^												
IVW	70	0.88 (0.81–0.95)	< 0.001	0.171	67	0.78 (0.72–0.85)	< 0.001	0.202	70	0.73 (0.57–0.94)	< 0.001	0.094
MR-Egger		0.43 (0.09–2.28)	0.386			0.56 (0.11–2.66)	0.502			0.90 (0.12–5.35)	0.915	

^a^ Multivariable MR analysis estimating the effect of regular glucosamine use on incident dementia, conditioning on vitamin supplement, chondroitin product intake, and osteoarthritis. All statistical tests were two-sided *P* < 0.05 was considered significant

In MVMR, the genetic liabilities for regular glucosamine, vitamin, chondroitin use, and osteoarthritis were evaluated. Use of glucosamine continued to have a significant effect on all-cause dementia (IVW odds ratio, 0.88; 95% CI, 0.81–0.95; *P* < 0.001), AD (IVW odds ratio, 0.78; 95% CI, 0.72–0.85; *P* < 0.001) and vascular dementia (IVW odds ratio, 0.73; 95% CI, 0.57–0.94; *P* < 0.001). These results align with those derived from the MVMR-Egger sensitivity analyses. Again, no horizontal pleiotropy was found in the MR-Egger intercept analysis. Details on the instruments used in MVMR can be found in Additional file 1: Table S14-15.

## Discussion

We observed that regular glucosamine use was related to a 15% decreased risk of all-cause dementia, 17% for AD, and 26% for vascular dementia in this large population-based study including 494,814 participants. These associations remained after adjusting for variables including sociodemographic factors, lifestyle behavior, comorbid conditions, medication, and other dietary conditions. Moreover, the beneficial effect of glucosamine use on AD seemed to be larger in participants aged below 60 years than in those aged above 60 years. The *APOE* genotype did not modify this association. In the MR analysis, we again observed protective causal effects of regular glucosamine use on dementia risk. Our findings were mostly consistent among various MR methods that made various assumptions regarding horizontal pleiotropy, demonstrating that horizontal pleiotropy is not probable to be a sufficient explanation for our findings.

We found that 19.0% of participants used glucosamine; this number is close to the 22.0% of the Australians over 45 who also take glucosamine [5]. Our findings are in line with a prior cross-sectional investigation that found glucosamine intake to be related to better cognitive function [18]. Glucosamine users had a higher reasoning score and faster reaction speed than non-users [18]. Furthermore, in a mouse model, glucosamine exerted a cognition-enhancing function [20], which implicated the beneficial impact of glucosamine use on dementia prevention.

Because glucosamine and chondroitin supplements are typically used simultaneously once daily [6], our observed relationships might be attributed to either of these supplements. To address this concern, a sensitivity analysis was conducted to test whether glucosamine alone (without chondroitin) could prevent dementia. No substantial change occurred in the sensitivity analyses. Thus, we speculate that glucosamine use might have a preventive role in the development of dementia, independent of chondroitin co-administration.

In our study, a stronger effect was found between glucosamine use and AD among participants aged below 60 years compared with those above 60 years. The weaker effect of glucosamine use in older participants may be related to the gradual atrophy of the hippocampus and the reduction of cortical density as the age increases, resulting in the reduction of brain cell membrane receptors and the decreased sensitivity to drugs [67]. This result underscores the age-modified connection between glucosamine use and dementia and emphasizes the importance of early prevention of dementia.

The protective association between glucosamine use and dementia may be explained by a few different processes. As a popular supplement that can pass through the blood–brain barrier, glucosamine may get to the hippocampus, striatum, and cortex [68, 69]. Meanwhile, several glucosamine transporters were identified in the brain [70]. For instance, glucose transporter 2 (GLUT2) was found in neurons and exhibited the greatest affinity for glucosamine [71, 72]. Intriguing evidence indicates that specific neuronal populations rely on GLUT2 to regulate glucose levels, thereby affecting their vulnerability to pathogenic mechanisms underlying AD [73, 74]. These studies highly support the important role of glucosamine on dementia. C-reactive protein, an indicator of systemic inflammation, was significantly lower in those who regularly took glucosamine, according to data from the National Health and Nutrition Examination Survey (NHANES) [8]. Animal studies also showed that glucosamine might suppress neuroinflammation [75], which is proved to increase the risk of dementia [76]. Furthermore, a prior research discovered that glucosamine might simulate a low-carbohydrate diet in mice through lowering glycolysis and enhancing amino acid catabolism [77]: consequently, glucosamine has been considered a mimicking agent for energy restriction [21]. Recent works demonstrated that a low-carbohydrate diet protects against the development of dementia [78, 79]. In addition, glucosamine could reverse the imbalanced gut microbiota [80]. Through the gut–brain axis, the gut microbiota modulates the brain functioning of the host and plays a significant role in dementia pathogenesis [81, 82]. Thus, glucosamine might have a beneficial effect on dementia pathology by regulating the gut microbiota. Other pathways may possibly be relevant and warrants further studies to explore the functional roles of glucosamine in dementia.

Our research had a number of advantages, such as a large number of participants and abundant data on dietary, health-related behaviors, and various factors that enabled us to examine the robustness of the findings and explore the effects of exposure in several subgroups. Furthermore, the MR analysis offered a superior method of obtaining somewhat less confounded estimates of causal associations that were not impacted by reverse causation or confounding. We admit that our research has limitations. Firstly, the “regular glucosamine use” was defined as self-reported at the baseline only, which might have changed in the follow-up period. Details on glucosamine use, such as dose and use duration, were not collected in the UK Biobank, which may weaken the study findings. Hence, further research that incorporates the glucosamine intake pattern and cross-validates the data on glucosamine for accuracy is required to delve into these connections. Secondly, UK Biobank did not record the adverse side effects participants suffered after using glucosamine. Nonetheless, glucosamine has been proved to be a safe supplementation for individuals with osteoarthritis due to its low risk of side effects including rare allergic reactions and gastrointestinal reactions [3]. Although people at high risk of diabetes showed reduced glucose tolerance after taking glucosamine [83, 84], studies have proved that in healthy people and diabetic patients, any oral dose of glucosamine will not affect the glucose metabolism and lipid status [85, 86]. Thirdly, in general, 20–100 imputed datasets are recommended, while in this study 5 datasets were imputed. Due to rather low proportions of missing data, we consider five imputed datasets to operate well. Fourthly, despite the SNPs we used were significantly correlated with the exposure, the genetic variants reflected only a modest portion of the overall variance in glucosamine intake, limiting them from being precise proxies of exposure. Given that we do not yet know how the genetic instruments work biologically, we cannot totally eliminate out breaches of the independence and exclusion restriction assumptions, especially with regard to pleiotropy [63]. Nevertheless, to infer reliable causal estimates, we used a variety of techniques, including Cochran's Q statistic, MR-PRESSO, weighted median, and MR-Egger. Fifthly, the interpretation of genetic liability of supplement use should be cautious as genetic predictors of glucosamine may capture participants with worse joint health [87]. We further adjusted osteoarthritis in the multivariable MR analysis to reduce bias. Sixthly, MR is a useful option for validating results; nevertheless, genetic variants reflect lifetime exposures rather than brief treatment modalities, which may create a bigger impact than a time-limited intervention [88]. Therefore, our findings should be taken cautiously, since they are hypothesis generating and warrant more clinical data to further investigate the connection between glucosamine intake and dementia. Seventhly, although the current definition for dementia was widely used in previous studies and the true positive rate for all-cause dementia collected in the UK Biobank was as high as 82.5% [89]; the true positive rates of Alzheimer’s disease and vascular dementia were lower than 75%. Thus, the results on the subtypes of dementia should be taken cautiously.

## Conclusions

Regular glucosamine use was associated with a lower risk of all-cause dementia, AD, and vascular dementia, based on data from the UK Biobank cohort and a mendelian randomization study. The potential implications of our findings for dementia prevention need additional confirmation in well-powered randomized controlled trials. We also recommend additional basic scientific research to investigate the underlying mechanisms.

## Supplementary Information

**Additional file 1:** **Table S1.** Disease definitions used in the UK Biobank study. **Table S2.** The numbers (percentages) of participants with missing covariates. **Table S3.** Results from sensitivity analyses for the relationship between regular glucosamine use and incident dementia. **Table S4.** GWAS summary statistics: source and description. **Table S5.** Summary information on glucosamine SNPs used as genetic instruments for the Mendelian randomization analyses. **Table S6.** Summary information on chondroitin SNPs used as genetic instruments for the Mendelian randomization analyses. **Table S7.** Summary information on vitamin supplement SNPs used as genetic instruments for the Mendelian randomization analyses. **Table S8.** Summary information on osteoarthritis SNPs used as genetic instruments for the Mendelian randomization analyses. **Table S9.** Potential confounders of exposures SNPs under the condition of *P* < 5 × 10^–8^ in the PhenoScanner database. **Table S10.** Summary information on all-cause dementia for the 9 genome-wide significant SNPs associated with glucosamine. **Table S11.** Summary information on Alzheimer’ disease for the 9 genome-wide significant SNPs associated with glucosamine. **Table S12.** Summary information on vascular dementia for the 9 genome-wide significant SNPs associated with glucosamine. **Table S13.** Results of MR Steiger direction test for glucosamine on dementia. **Table S14.** Independent instruments used for multivariable MR. Table S15. Summary information on dementia for the genome-wide significant SNPs associated with multivariable Instruments. **Figure S1.** Forest plot for the relationship of regular glucosamine use with incident dementia. **Figure S2.** Leave-one-out analyses for SNPs associated with regular glucosamine use on incident dementia.

## Data Availability

Data are available in a public, open access repository. Data from the UK Biobank (https://www.ukbiobank.ac.uk/) are available to researchers on application. The application number of this research is 55794.

## Abbreviations

MR: Mendelian randomization
AD: Alzheimer’s disease
GWAS: Genome-wide association studies
HR: Hazard ratios
MV-IVW: Multivariable inverse variance weighted
ICD: International Classification of Diseases
TDI: Townsend Deprivation Index
BMI: Body mass index
NSAIDs: Non-steroidal anti-inflammatory drugs
CIs: Confidence intervals
SNPs: Single-nucleotide polymorphisms

## References

[1] Livingston G, Huntley J, Sommerlad A, Ames D, Ballard C, Banerjee S, Brayne C, Burns A, Cohen-Mansfield J, Cooper C, Costafreda SG, Dias A, Fox N, Gitlin LN, Howard R, Kales HC, Kivimaki M, Larson EB, Ogunniyi A, Orgeta V, Ritchie K, Rockwood K, Sampson EL, Samus Q, Schneider LS, Selbaek G, Teri L, Mukadam N (2020). Dementia prevention, intervention, and care: 2020 report of the Lancet Commission. Lancet.

[2] NHS England. Dementia. https://www.england.nhs.uk/mental/health/dementia (Accessed 2 Sept 2022).

[3] Jordan K, Arden N, Doherty M, Bannwarth B, Bijlsma J, Dieppe P, Gunther K, Hauselmann H, Herrero-Beaumont G, Kaklamanis P (2003). EULAR Recommendations 2003: an evidence based approach to the management of knee osteoarthritis: Report of a Task Force of the Standing Committee for International Clinical Studies Including Therapeutic Trials (ESCISIT). Ann Rheum Dis.

[4] Barnes PM, Bloom B, Nahin RL. Complementary and alternative medicine use among adults and children: United States, 2007. 2008. 19361005

[5] Sibbritt D, Adams J, Lui CW, Broom A, Wardle J. Who uses glucosamine and why? A study of 266,848 Australians aged 45 years and older. 2012. 10.1371/journal.pone.0041540 PMC3408465 22859995

[6] Clegg DO, Reda DJ, Harris CL, Klein MA, O’Dell JR, Hooper MM, Bradley JD, Bingham CO III, Weisman MH, Jackson CG. Glucosamine, chondroitin sulfate, and the two in combination for painful knee osteoarthritis. N Engl J Med. 2006;354(8):795–808. 10.1056/NEJMoa052771 16495392

[7] Wilkens P, Scheel IB, Grundnes O, Hellum C, Storheim K (2010). Effect of glucosamine on pain-related disability in patients with chronic low back pain and degenerative lumbar osteoarthritis: a randomized controlled trial. JAMA.

[8] Kantor ED, Lampe JW, Vaughan TL, Peters U, Rehm CD, White E (2012). Association between use of specialty dietary supplements and C-reactive protein concentrations. Am J Epidemiol.

[9] Mantovani A, Allavena P, Sica A, Balkwill F (2008). Cancer-related inflammation. Nature.

[10] Willerson JT, Ridker PM. Inflammation as a cardiovascular risk factor. Circulation. 2004;109 (21_suppl_1):II-2-II-10. 10.1161/01.CIR.0000129535.04194.38 15173056

[11] Lee DH, Cao C, Zong X, Zhang X, O’Connell K, Song M, Wu K, Du M, Cao Y, Giovannucci EL. Glucosamine and Chondroitin Supplements and Risk of Colorectal Adenoma and Serrated PolypGlucosamine, Chondroitin, and Colorectal Adenoma. Cancer Epidemiol Biomarkers Prev. 2020;29(12):2693–701. 10.1158/1055-9965.EPI-20-0805 PMC7710617 33055203

[12] Kantor ED, Newton CC, Giovannucci EL, McCullough ML, Campbell PT, Jacobs EJ (2018). Glucosamine use and risk of colorectal cancer: results from the Cancer Prevention Study II Nutrition Cohort. Cancer Causes Control.

[13] Li G, Zhang X, Liu Y, Zhang J, Li L, Huang X, Thabane L, Lip GYH. Relationship between glucosamine use and the risk of lung cancer: data from a nationwide prospective cohort study. Eur Respir J. 2022;59(3):2101399. 10.1183/13993003.01399-2021. 10.1183/13993003.01399-2021 34326189

[14] King DE, Xiang J (2020). Glucosamine/chondroitin and mortality in a US NHANES cohort. J Am Board Fam Med.

[15] Ma H, Li X, Sun D, Zhou T, Ley SH, Gustat J, Heianza Y, Qi L. Association of habitual glucosamine use with risk of cardiovascular disease: prospective study in UK Biobank. BMJ. 2019;365:l1628. 10.1136/bmj.l1628. 10.1136/bmj.l1628 PMC6515311 31088786

[16] Ma H, Li X, Zhou T, Sun D, Liang Z, Li Y, Heianza Y, Qi L (2020). Glucosamine use, inflammation, and genetic susceptibility, and incidence of type 2 diabetes: a prospective study in UK Biobank. Diabetes Care.

[17] Bell GA, Kantor ED, Lampe JW, Shen DD, White E (2012). Use of glucosamine and chondroitin in relation to mortality. Eur J Epidemiol.

[18] Nevado-Holgado AJ, Kim C-H, Winchester L, Gallacher J, Lovestone S (2016). Commonly prescribed drugs associate with cognitive function: a cross-sectional study in UK Biobank. BMJ Open.

[19] Araújo AR, Castro VIB, Reis RL, Pires RA (2021). Glucosamine and Its Analogues as Modulators of Amyloid-β Toxicity. ACS Med Chem Lett.

[20] Chou L-Y, Chao Y-M, Peng Y-C, Lin H-C, Wu Y-L (2020). Glucosamine enhancement of BDNF expression and animal cognitive function. Molecules.

[21] Nikolai S, Pallauf K, Huebbe P, Rimbach G (2015). Energy restriction and potential energy restriction mimetics. Nutr Res Rev.

[22] Shang X, Hill E, Zhu Z, Liu J, Ge Z, Wang W, He M. Macronutrient Intake and Risk of Dementia in Community-Dwelling Older Adults: A Nine-Year Follow-Up Cohort Study. J Alzheimer’s Dis. 2022;85(2):791–804. 10.3233/JAD-215042 34864666

[23] Gentreau M, Chuy V, Féart C, Samieri C, Ritchie K, Raymond M, Berticat C, Artero S. Refined carbohydrate-rich diet is associated with long-term risk of dementia and Alzheimer’s disease in apolipoprotein E ε4 allele carriers. Alzheimers Dement. 2020;16(7):1043–53. 10.1002/alz.12114 32506713

[24] Jhelum P, Radhakrishnan M, Paul A, Dey SK, Kamle A, Kumar A, Sharma A, Chakravarty S. Neuroprotective and Proneurogenic Effects of Glucosamine in an Internal Carotid Artery Occlusion Model of Ischemia. NeuroMol Med. 2021;1–6. 10.1007/s12017-021-08697-5 34837638

[25] Ma X, Li H, He Y, Hao J (2017). The emerging link between O-GlcNAcylation and neurological disorders. Cell Mol Life Sci.

[26] Akan I, Olivier-Van Stichelen S, Bond MR, Hanover JA. Nutrient-driven O-GlcNAc in proteostasis and neurodegeneration. J Neurochem. 2018;144(1):7–34. 10.1111/jnc.14242. 10.1111/jnc.14242 PMC5735008 29049853

[27] Lawlor DA, Harbord RM, Sterne JA, Timpson N, Davey Smith G (2008). Mendelian randomization: using genes as instruments for making causal inferences in epidemiology. Stat Med.

[28] Sudlow C, Gallacher J, Allen N, Beral V, Burton P, Danesh J, Downey P, Elliott P, Green J, Landray M, Liu B, Matthews P, Ong G, Pell J, Silman A, Young A, Sprosen T, Peakman T, Collins R (2015). UK biobank: an open access resource for identifying the causes of a wide range of complex diseases of middle and old age. PLoS Med.

[29] Li ZH, Zhong WF, Liu S, Kraus VB, Zhang YJ, Gao X, Lv YB, Shen D, Zhang XR, Zhang PD, Huang QM, Chen Q, Wu XB, Shi XM, Wang D, Mao C (2020). Associations of habitual fish oil supplementation with cardiovascular outcomes and all cause mortality: evidence from a large population based cohort study. BMJ.

[30] Li Z-H, Gao X, Chung VC, Zhong W-F, Fu Q, Lv Y-B, Wang Z-H, Shen D, Zhang X-R, Zhang P-D (2020). Associations of regular glucosamine use with all-cause and cause-specific mortality: a large prospective cohort study. Ann Rheum Dis.

[31] Calvin CM, Wilkinson T, Starr JM, Sudlow C, Hagenaars SP, Harris SE, Schnier C, Davies G, Fawns-Ritchie C, Gale CR, Gallacher J, Deary IJ. Predicting incident dementia 3–8 years after brief cognitive tests in the UK Biobank prospective study of 500,000 people. Alzheimer’s Dementia. 2019;15(12):1546–57. 10.1016/j.jalz.2019.07.014 31619348

[32] Wilkinson T, Ly A, Schnier C, Rannikmäe K, Bush K, Brayne C, Quinn TJ, Sudlow CLM. Identifying dementia cases with routinely collected health data: A systematic review. Alzheimer’s Dementia. 2018;14(8):1038–51. 10.1016/j.jalz.2018.02.016 PMC6105076 29621480

[33] Bycroft C, Freeman C, Petkova D, Band G, Elliott LT, Sharp K, Motyer A, Vukcevic D, Delaneau O, O’Connell J, Cortes A, Welsh S, Young A, Effingham M, McVean G, Leslie S, Allen N, Donnelly P, Marchini J. The UK Biobank resource with deep phenotyping and genomic data. Nature. 2018;562(7726):203–9. 10.1038/s41586-018-0579-z PMC6786975 30305743

[34] Mattsson N, Groot C, Jansen WJ, Landau SM, Villemagne VL, Engelborghs S, Mintun MM, Lleo A, Molinuevo JL, Jagust WJ, Frisoni GB, Ivanoiu A, Chételat G, Resende de Oliveira C, Rodrigue KM, Kornhuber J, Wallin A, Klimkowicz-Mrowiec A, Kandimalla R, Popp J, Aalten PP, Aarsland D, Alcolea D, Almdahl IS, Baldeiras I, van Buchem MA, Cavedo E, Chen K, Cohen AD, Förster S, Fortea J, Frederiksen KS, Freund-Levi Y, Gill KD, Gkatzima O, Grimmer T, Hampel H, Herukka SK, Johannsen P, van Laere K, de Leon MJ, Maier W, Marcusson J, Meulenbroek O, Møllergård HM, Morris JC, Mroczko B, Nordlund A, Prabhakar S, Peters O, Rami L, Rodríguez-Rodríguez E, Roe CM, Rüther E, Santana I, Schröder J, Seo SW, Soininen H, Spiru L, Stomrud E, Struyfs H, Teunissen CE, Verhey FRJ, Vos SJB, van Waalwijk van Doorn LJC, Waldemar G, Wallin ÅK, Wiltfang J, Vandenberghe R, Brooks DJ, Fladby T, Rowe CC, Drzezga A, Verbeek MM, Sarazin M, Wolk DA, Fleisher AS, Klunk WE, Na DL, Sánchez-Juan P, Lee DY, Nordberg A, Tsolaki M, Camus V, Rinne JO, Fagan AM, Zetterberg H, Blennow K, Rabinovici GD, Hansson O, van Berckel BNM, van der Flier WM, Scheltens P, Visser PJ, Ossenkoppele R. Prevalence of the apolipoprotein E ε4 allele in amyloid β positive subjects across the spectrum of Alzheimer's disease. Alzheimer’s Dementia. 2018;14(7):913–924. 10.1016/j.jalz.2018.02.009 29601787

[35] Querfurth HW, LaFerla FM. Alzheimer’s disease. N Engl J Med. 2010;362(4):329–44. 10.1056/NEJMra0909142 20107219

[36] Garland A, Fransoo R, Olafson K, Ramsey C, Chateau, D. The Epidemiology and Outcomes of Critical Illness in Manitoba. 2012.

[37] Gutiérrez-Sacristán A, Bravo À, Giannoula A, Mayer MA, Sanz F, Furlong LI. comoRbidity: an R package for the systematic analysis of disease comorbidities. Bioinformatics. 2018;34(18):3228–30. 10.1093/bioinformatics/bty315. 10.1093/bioinformatics/bty315 PMC6137966 29897411

[38] Jarman B, Townsend P, Carstairs V (1991). Deprivation indices. BMJ.

[39] World Health Organization t. Global recommendations on physical activity for health. World Health Organization: 2010. https://www.who.int/publications/i/item/9789241599979. 26180873

[40] Association of habitual glucosamine use with risk of cardiovascular disease: prospective study in UK Biobank. BMJ. 2019;365. 10.1136/bmj.l1628 PMC6515311 31088786

[41] Li ZH, Gao X, Chung VC, Zhong WF, Mao C. Associations of regular glucosamine use with all-cause and cause-specific mortality: a large prospective cohort study. Ann Rheum Dis. 2020;annrheumdis-2020–217176. 10.1136/annrheumdis-2020-217176 PMC7286049 32253185

[42] Hagenaars SP, Harris SE, Davies G, Hill WD, Liewald DC, Ritchie SJ, Marioni RE, Fawns-Ritchie C, Cullen B, Malik R. Shared genetic aetiology between cognitive functions and physical and mental health in UK Biobank (*N*= 112 151) and 24 GWAS consortia. Mol Psychiatry. 2016;21(11):1624–32. 10.1038/mp.2015.225 PMC5078856 26809841

[43] Davies G, Marioni RE, Liewald DC, Hill WD, Hagenaars SP, Harris SE, Ritchie SJ, Luciano M, Fawns-Ritchie C, Lyall D. Genome-wide association study of cognitive functions and educational attainment in UK Biobank (*N*= 112 151). Mol Psychiatry. 2016;21(6):758–67. 10.1038/mp.2016.45 PMC4879186 27046643

[44] Lyall DM, Cullen B, Allerhand M, Smith DJ, Mackay D, Evans J, Anderson J, Fawns-Ritchie C, McIntosh AM, Deary IJ (2016). Cognitive test scores in UK Biobank: data reduction in 480,416 participants and longitudinal stability in 20,346 participants. PLoS ONE.

[45] Van Buuren S, Groothuis-Oudshoorn K (2011). mice: Multivariate imputation by chained equations in R. J Stat Softw.

[46] Van Buuren S. Flexible imputation of missing data. CRC press: 2018. https://www.jstatsoft.org/htaccess.php?volume=093&type=b&issue=01&filename=paper.

[47] Schoenfeld D (1982). Partial residuals for the proportional hazards regression model. Biometrika.

[48] Didelez V, Sheehan N (2007). Mendelian randomization as an instrumental variable approach to causal inference. Stat Methods Med Res.

[49] Angrist JD, Imbens GW, Rubin DB (1996). Identification of causal effects using instrumental variables. J Am Stat Assoc.

[50] Sudlow C, Gallacher J, Allen N, Beral V, Burton P, Danesh J, Downey P, Elliott P, Green J, Landray M (2015). UK biobank: an open access resource for identifying the causes of a wide range of complex diseases of middle and old age. PLoS Med.

[51] Neale Lab. GWAS round 2. 2018. http://www.nealelab.is/uk-biobank/. Accessed 2 Sept 2022.

[52] Lambert J-C, Ibrahim-Verbaas CA, Harold D, Naj AC, Sims R, Bellenguez C, Jun G, DeStefano AL, Bis JC, Beecham GW. Meta-analysis of 74,046 individuals identifies 11 new susceptibility loci for Alzheimer’s disease. Nat Genet. 2013;45(12):1452–8. 10.1038/ng.2802 PMC3896259 24162737

[53] FinnGen documentation of R4 release, 2020. https://finngen.gitbook.io/documentation/. Accessed 2 Sept 2022.

[54] Hemani G, Zheng J, Elsworth B, Wade KH, Haberland V, Baird D, et al. The MR-Base platform supports systematic causal inference across the human phenome. elife. 2018;7:e34408. 10.7554/eLife.34408. 10.7554/eLife.34408 PMC5976434 29846171

[55] Meddens SFW, de Vlaming R, Bowers P, Burik CA, Linnér RK, Lee C, Okbay A, Turley P, Rietveld CA, Fontana MA (2021). Genomic analysis of diet composition finds novel loci and associations with health and lifestyle. Mol Psychiatry.

[56] Palmer TM, Lawlor DA, Harbord RM, Sheehan NA, Tobias JH, Timpson NJ, Smith GD, Sterne JA (2012). Using multiple genetic variants as instrumental variables for modifiable risk factors. Stat Methods Med Res.

[57] Loh P, Kichaev G, Gazal S, Schoech A, Price A (2018). Mixed-model association for biobank-scale datasets. Nat Genet.

[58] Bowden J, Davey Smith G, Burgess S (2015). Mendelian randomization with invalid instruments: effect estimation and bias detection through Egger regression. Int J Epidemiol.

[59] Bowden J, Davey Smith G, Haycock PC, Burgess S (2016). Consistent estimation in Mendelian randomization with some invalid instruments using a weighted median estimator. Genet Epidemiol.

[60] Bowden J, Del Greco MF, Minelli C, Davey Smith G, Sheehan N, Thompson J. A framework for the investigation of pleiotropy in two‐sample summary data Mendelian randomization. Stat Med. 2017;36(11):1783–1802. 10.1002/sim.7221 PMC5434863 28114746

[61] Verbanck M, Chen C-Y, Neale B, Do R (2018). Detection of widespread horizontal pleiotropy in causal relationships inferred from Mendelian randomization between complex traits and diseases. Nat Genet.

[62] Bowden J, Del Greco MF, Minelli C, Zhao Q, Lawlor DA, Sheehan NA, Thompson J, Davey Smith G. Improving the accuracy of two-sample summary-data Mendelian randomization: moving beyond the NOME assumption. Int J Epidemiol. 2019;48(3):728–742. 10.1093/ije/dyy258 PMC6659376 30561657

[63] Hemani G, Bowden J, Davey Smith G (2018). Evaluating the potential role of pleiotropy in Mendelian randomization studies. Hum Mol Genet.

[64] Hemani G, Tilling K, Davey Smith G (2017). Orienting the causal relationship between imprecisely measured traits using GWAS summary data. PLoS Genet.

[65] Burgess S, Thompson SG (2015). Multivariable Mendelian randomization: the use of pleiotropic genetic variants to estimate causal effects. Am J Epidemiol.

[66] Rees JM, Wood AM, Burgess S (2017). Extending the MR-Egger method for multivariable Mendelian randomization to correct for both measured and unmeasured pleiotropy. Stat Med.

[67] Spreng RN, Turner GR. Structure and function of the aging brain. 2019.

[68] Popov N (1985). Effects of D-galactosamine and D-glucosamine on retention performance of a brightness discrimination task in rats. Biomed Biochim Acta.

[69] Setnikar I, Rovati LC (2001). Absorption, distribution, metabolism and excretion of glucosamine sulfate. Arzneimittelforschung.

[70] Jurcovicova J (2014). Glucose transport in brain-effect of inflammation. Endocr Regul.

[71] Mardones L, Ormazabal V, Romo X, Jaña C, Binder P, Peña E, Vergara M, Zúñiga FA (2011). The glucose transporter-2 (GLUT2) is a low affinity dehydroascorbic acid transporter. Biochem Biophys Res Commun.

[72] Uldry M, Ibberson M, Hosokawa M, Thorens B (2002). GLUT2 is a high affinity glucosamine transporter. FEBS Lett.

[73] Plaschke K, Kopitz J, Siegelin M, Schliebs R, Salkovic-Petrisic M, Riederer P, Hoyer S. Insulin-resistant brain state after intracerebroventricular streptozotocin injection exacerbates Alzheimer-like changes in Tg2576 AβPP-overexpressing mice. J Alzheimer’s Dis. 2010;19(2):691–704. 10.3233/JAD-2010-1270 20110613

[74] Knezovic A, Loncar A, Homolak J, Smailovic U, Osmanovic Barilar J, Ganoci L, Bozina N, Riederer P, Salkovic-Petrisic M (2017). Rat brain glucose transporter-2, insulin receptor and glial expression are acute targets of intracerebroventricular streptozotocin: risk factors for sporadic Alzheimer’s disease?. J Neural Transm.

[75] Lee Y, Lee S, Park J-W, Hwang J-S, Kim S-M, Lyoo IK, Lee C-J, Han I-O (2018). Hypoxia-induced neuroinflammation and learning–memory impairments in adult zebrafish are suppressed by glucosamine. Mol Neurobiol.

[76] Contreras JA, Aslanyan V, Albrecht DS, Mack WJ. Initiative, A. s. D. N.; Pa, J., Higher baseline levels of CSF inflammation increase risk of incident mild cognitive impairment and Alzheimer's disease dementia. Alzheimer’s Dementia 2022;14(1):e12346. 10.1002/dad2.12346 PMC9484791 36187197

[77] Weimer S, Priebs J, Kuhlow D, Groth M, Priebe S, Mansfeld J, Merry TL, Dubuis S, Laube B, Pfeiffer AF (2014). D-Glucosamine supplementation extends life span of nematodes and of ageing mice. Nat Commun.

[78] Reger MA, Henderson ST, Hale C, Cholerton B, Baker LD, Watson GS, Hyde K, Chapman D, Craft S (2004). Effects of β-hydroxybutyrate on cognition in memory-impaired adults. Neurobiol Aging.

[79] Rebello CJ, Keller JN, Liu AG, Johnson WD, Greenway FL (2015). Pilot feasibility and safety study examining the effect of medium chain triglyceride supplementation in subjects with mild cognitive impairment: A randomized controlled trial. BBA clinical.

[80] Shmagel A, Demmer R, Knights D, Butler M, Langsetmo L, Lane NE, Ensrud K (2019). The effects of glucosamine and chondroitin sulfate on gut microbial composition: a systematic review of evidence from animal and human studies. Nutrients.

[81] Chen C, Ahn EH, Kang SS, Liu X, Alam A, Ye K. Gut dysbiosis contributes to amyloid pathology, associated with C/EBPβ/AEP signaling activation in Alzheimer’s disease mouse model. Sci Adv. 2020;6(31):eaba0466. 10.1126/sciadv.aba0466 PMC7439296 32832679

[82] Wang X, Sun G, Feng T, Zhang J, Huang X, Wang T, Xie Z, Chu X, Yang J, Wang H (2019). Sodium oligomannate therapeutically remodels gut microbiota and suppresses gut bacterial amino acids-shaped neuroinflammation to inhibit Alzheimer’s disease progression. Cell Res.

[83] Pham T, Cornea A, Jenkins A, Blick KE, Scofield RH (2007). Oral glucosamine in doses used to treat osteoarthritis worsens insulin resistance. Am J Med Sci.

[84] Biggee BA, Blinn CM, Nuite M, Silbert JE, McAlindon TE (2007). Effects of oral glucosamine sulphate on serum glucose and insulin during an oral glucose tolerance test of subjects with osteoarthritis. Ann Rheum Dis.

[85] Albert SG, Fishman Oiknine R, Parseghian S, Mooradian AD, Haas MJ, McPherson T (2007). The effect of glucosamine on serum HDL cholesterol and apolipoprotein AI levels in people with diabetes. Diabetes Care.

[86] Simon R, Marks V, Leeds A, Anderson J (2011). A comprehensive review of oral glucosamine use and effects on glucose metabolism in normal and diabetic individuals. Diabetes Metab Res Rev.

[87] Wu Y, Byrne E, Zheng Z, Kemper K, Yengo L, Mallett A, Yang J, Visscher P, Wray N (2019). Genome-wide association study of medication-use and associated disease in the UK Biobank. Nat Commun.

[88] Davies NM, Holmes MV, Davey Smith G. Reading Mendelian randomisation studies: a guide, glossary, and checklist for clinicians. BMJ. 2018;362:k601. 10.1136/bmj.k601. 10.1136/bmj.k601 PMC6041728 30002074

[89] Wilkinson T, Schnier C, Bush K, Rannikmäe K, Henshall DE, Lerpiniere C, Allen NE, Flaig R, Russ TC, Bathgate D, Pal S, O’Brien JT, Sudlow CLM. Identifying dementia outcomes in UK Biobank: a validation study of primary care, hospital admissions and mortality data. Eur J Epidemiol. 2019;34(6):557–65. 10.1007/s10654-019-00499-1 PMC6497624 30806901

