# Correction: Association of regular glucosamine use with incident dementia: evidence from a longitudinal cohort and Mendelian randomization study

**DOI:** 10.1186/s12916-023-02892-w

**Published:** 2023-05-08

**Authors:** Jiazhen Zheng, Can Ni, Yingchai Zhang, Jinghan Huang, Daniel Nyarko Hukportie, Buwen Liang, Shaojun Tang

**Affiliations:** 1 Bioscience and Biomedical Engineering Thrust, Systems Hub, The Hong Kong University of Science and Technology (Guangzhou), Guangzhou, Guangdong China; 2 Department of Medicine and Therapeutics, Prince of Wales Hospital, The Chinese University of Hong Kong, Sha Tin, New Territories, Hong Kong, SAR China; 3 Biomedical Genetics Section, School of Medicine, Boston University, Boston, USA; 4 Department of Chemical Pathology, Faculty of Medicine, The Chinese University of Hong Kong, Hong Kong, SAR China; 5 Department of Epidemiology, School of Public Health, (Guangdong Provincial Key Laboratory of Tropical Disease Research), Southern Medical University, Guangzhou, China; 6 Division of Emerging Interdisciplinary Areas, The Hong Kong University of Science and Technology, Clear Water Bay, Hong Kong, SAR China

**Correction: BMC Med 21, 114 (2023)**

**https://doi.org/10.1186/s12916-023-02816-8**

The original article [1] mistakenly listed the UKB application ID number as 55794. The correct ID number is 97089.

## References

[1] Zheng J (2023). Association of regular glucosamine use with incident dementia: evidence from a longitudinal cohort and Mendelian randomization study. BMC Med.

