# Supplementary material for: Association of regular glucosamine use with incident dementia: evidence from a longitudinal cohort and Mendelian randomization study
Source: BMC Med. 2023 Mar 29;21:114. doi: 10.1186/s12916-023-02816-8 (PMC10052856; doi:10.1186/s12916-023-02816-8)
Supplement: Supplementary file 1 — Additional file 1: Table S1. Disease definitions used in the UK Biobank study. Table S2. The numbers (percentages) of participants with missing covariates. Table S3. Results from sensitivity analyses for the relationship between regular glucosamine use and incident dementia. Table S4. GWAS summary statistics: source and description. Table S5. Summary information on glucosamine SNPs used as genetic instruments for the Mendelian randomization analyses. Table S6. Summary information on chondroitin SNPs used as genetic instruments for the Mendelian randomization analyses. Table S7. Summary information on vitamin supplement SNPs used as genetic instruments for the Mendelian randomization analyses. Table S8. Summary information on osteoarthritis SNPs used as genetic instruments for the Mendelian randomization analyses. Table S9. Potential confounders of exposures SNPs under the condition of P < 5 × 10^–8^ in the PhenoScanner database. Table S10. Summary information on all-cause dementia for the 9 genome-wide significant SNPs associated with glucosamine. Table S11. Summary information on Alzheimer’ disease for the 9 genome-wide significant SNPs associated with glucosamine. Table S12. Summary information on vascular dementia for the 9 genome-wide significant SNPs associated with glucosamine. Table S13. Results of MR Steiger direction test for glucosamine on dementia. Table S14. Independent instruments used for multivariable MR. Table S15. Summary information on dementia for the genome-wide significant SNPs associated with multivariable Instruments. Figure S1. Forest plot for the relationship of regular glucosamine use with incident dementia. Figure S2. Leave-one-out analyses for SNPs associated with regular glucosamine use on incident dementia. [file 12916_2023_2816_MOESM1_ESM.docx]

**Table S1. Disease definitions used in the UK Biobank study**

| Disease | UK Biobank Self Report Fields and Codes | ICD 9 | ICD 10 |
| --- | --- | --- | --- |
| All-cause dementia | Field 20002: Code 1263 | 290.2, 290.3, 290.4, 291.2, 294.1, 331.0, 331.1, 331.2, 331.5 | A81.0, F00, F00.0, F00.1, F00.2, F00.9, F01, F01.0, F01.1, F01.2, F01.3, F01.8, F01.9, F02, F02.0, F02.1, F02.2, F02.3, F02.4, F02.8, F03, F05.1, F10.6, G30, G30.0, G30.1, G30.8, G30.9, G31.0, G31.1, G31.8, I67.3 |
| Alzheimer's disease | - | 331.0 | F00, F00.0, F00.1, F00.2, F00.9, G30, G30.0, G30.1, G30.8, G30.9 |
| Vascular dementia | - | 290.4 | F01, F01.0, F01.1, F01.2, F01.3, F01.8, F01.9, I67.3 |

**Table S2. The numbers (percentages) of participants with missing covariates**

| **Covariates** | **n** | **%** |
| --- | --- | --- |
| Household income | 69,174 | 13.98 |
| Memory | 19,741 | 3.93 |
| Vegetable consumption | 9124 | 1.84 |
| Fruit consumption | 6,491 | 1.31 |
| Reaction time | 5,694 | 1.13 |
| Physical activity | 3,775 | 0.76 |
| Body mass index | 2,231 | 0.45 |
| Ethnicity | 1,622 | 0.33 |
| Smoking status | 1,621 | 0.33 |
| Diabetes | 1,266 | 0.26 |
| Townsend Deprivation Index | 608 | 0.12 |

| **Sensitivity analysis** | **Model 2*** | |
| --- | --- | --- |
|  | **HR (95% CI)** | **P value** |
| **All-cause dementia** |  |  |
| Performing competing risk analysis ^a^ | 0.84 (0.75-0.94) | <0.001 |
| Excluding participants taking chondroitin ^b^ | 0.85 (0.77-0.95) | 0.003 |
| Excluding participants with missing covariate data ^c^ | 0.89 (0.80-0.99) | 0.030 |
| Excluding participants who developed outcomes during the first two years of follow-up ^d^ | 0.85 (0.76-0.94) | 0.003 |
| Adding cases retrieved from primary care data using Read Codes ^e^ | 0.86 (0.80-0.92) | <0.001 |
| **Alzheimer’s disease** |  |  |
| Performing competing risk analysis ^f^ | 0.82 (0.70-0.97) | 0.011 |
| Excluding participants taking chondroitin ^b^ | 0.83 (0.70-0.98) | 0.029 |
| Excluding participants with missing covariate data ^c^ | 0.82 (0.68-0.98) | 0.023 |
| Excluding participants who developed outcomes during the first two years of follow-up ^g^ | 0.82 (0.69-0.98) | 0.019 |
| Adding cases retrieved from primary care data using Read Codes ^h^ | 0.83 (0.74-0.93) | <0.001 |
| **Vascular dementia** |  |  |
| Performing competing risk analysis ^i^ | 0.74 (0.58-0.96) | 0.026 |
| Excluding participants taking chondroitin ^b^ | 0.76 (0.59-0.97) | 0.028 |
| Excluding participants with missing covariate data ^c^ | 0.77 (0.59-0.99) | 0.046 |
| Excluding participants who developed outcomes during the first two years of follow-up ^j^ | 0.73 (0.56-0.95) | 0.021 |
| Adding cases retrieved from primary care data using Read Codes ^k^ | 0.74 (0.62-0.92) | 0.003 |

**Table S3. Results from sensitivity analyses for the relationship between regular glucosamine use and incident dementia.**HR: hazard ratio; CI: confidence interval. *Model 2: adjusted for age, sex, ethnicity, education, Townsend Deprivation Index, household income, body mass index, fruit consumption, vegetable consumption, smoking status, alcohol consumption, physical activity, health condition, chondroitin use, aspirin use, non-aspirin NSAID use, vitamin supplementation, mineral and other dietary supplementation, memory and reaction time.

^a^ There were 19,082 deaths as competing events for all-cause dementia.

^b^ There were 6,432 chondroitin users excluded for analyses.

^c^ There were 71,720 participants with missing covariate data excluded for analyses.

^d^ There were 405 participants who developed outcomes during the first two years of follow-up excluded for analyses.

^e^ 990 all- cause dementia cases were retrieved from primary care data using Read Codes

^f^ There were 19,654 deaths as competing events for Alzheimer’s disease.

^g^ There were 143 participants who developed outcomes during the first two years of follow-up excluded for analyses.

^h^ 493 alzheimer’s disease cases were retrieved from primary care data using Read Codes

^i^ There were 19,763 deaths as competing events for vascular dementia.

^j^ There were 90 participants who developed outcomes during the first two years of follow-up excluded for analyses.

^k^ 215 vascular dementia cases were retrieved from primary care data using Read Codes

**Supplemental Methods.**

***APOE* genotyping**

UK Biobank participants have their genotypes determined using UK BiLEVE or UK Biobank Axiom. After single-nucleotide polymorphism (SNP) and sample quality controls, the UK Biobank imputed directly genotyped data using from UK 10K haplotype, 1000 Genomes Phase 3, and Haplotype Reference Consortium reference panels. The *APOE* genotype was determined by two SNPs which include rs429358 and rs7412. We categorized the participants into *APOE* ε4 noncarriers (−/−) and carriers (+/− or +/+) since APOE ε4 is a known genetic risk factor for dementia and AD that mostly affects Aβ metabolism.

**Elixhauser Comorbidity Index**

The Elixhauser Comorbidity Index (ECI) is a means of categorizing comorbidities in patients based on the ICD-10 diagnosis codes; the most recent version has 31 categories. ICD-10 diagnosis codes of the study participants were mapped to ECI disease categories using the R package comorbidity. For each participant, ECI categories were scored as 0 or 1 according to the absence (0) or presence (1) of corresponding ICD-10 codes. Participants are given an ECI score that is the summation of all of their individual ECI disease category scores; the larger the score, the greater number of comorbidities a given individual has.

**Two-sample MR study design.**

For causal estimates from MR studies to be valid, three assumptions must be met: (1) the genetic variants are strongly associated with the exposure, (2) the genetic variants are not associated with any potential confounder of the exposure–outcome association and (3) the variants do not affect outcome independently of exposure. We first performed a two-sample single variable MR to assess the association of regular glucosamine use with dementia risk. Multivariable MR was used to further estimate the direct effect of glucosamine on dementia, independent of vitamin, chondroitin supplements and osteoarthritis.

**Data sources and instruments.**

Summary statistics for dietary supplements were obtained from UK Biobank (UKB), from which well-powered GWAS data was available. We used GWAS summary statistics of clinical factors based on 361,194 white-British participants released by the Neale Lab (<http://www.nealelab.is/uk-biobank>). Cohort measured habitual dietary intake using touch screen questionnaire. We obtained summary data from 360,016 participants with glucosamine, 361141 with chondroitin data, 54,162 with vitamin supplement data and with 462,933 osteoarthritis data (Table S4). All single-nucleotide polymorphisms (SNPs) selected for inclusion or exclusion in univariable MR analysis are presented in Tables S5–11.

**Sample independence.**

Participant overlap in samples used to estimate genetic associations between exposures and outcomes can increase weak instrument bias in MR analyses. Most of the recent meta-analyses of GWAS compute effect estimates by pooling UKB data sets with previously available data sets, and the existence of any overlapping samples in exposure and outcome datasets could bias the effect estimates toward the confounded observational estimates. We derived the summary data of Alzheimer from the International Genomics of Alzheimer’s Project Consortium (IGAP), which was a sizeable two-stage research based on GWASs of Alzheimer without UKB samples. The summary data of all-cause dementia and vascular dementia was derived from the FinnGen, which was a genomic data source combined with phenotype data collected by national health registries, including extensive longitudinal registry data available on most of the Finns . Since the data we used did not contain samples from the UKB, the overlap in our analyses of glucosamine use on dementia was negligible.

**Selection of the genetic instrumental variables.**

We selected all relevant SNVs identified in each GWASs as having reached selection threshold P less than 5 × 10^−8^ (5 × 10^−6^ for vitamin supplement) and being uncorrelated (10,000 kilobase pairs apart and R^2^ ≤.001). We obtained SNV effects and corresponding standard errors from the exposure and outcome GWASs . To avoid potential confounding, we investigated each instrument SNP in the PhenoScanner GWAS database to assess any previous associations (P<5×10^−8^) with plausible confounders (that is, alcohol consumption, smoking status, physical activity and education) . To meet the assumption that requires instruments to be associated with the outcome only through exposure, we excluded SNPs strongly associated with the outcome. We harmonized exposure and outcome data, removed palindromic SNPs with intermediate allele frequencies, and estimated the F parameter to evaluate instrument strength . We applied Steiger filtering to the harmonized data to identify and remove those SNPs exhibiting reverse causation by the test metric. The observed variance of the outcome exceeded the observed variance of the exposure explained by the SNPs (eTables in the Supplement).

**Testing instrument strength and statistical power.**

Testing instrument strength and statistical power. The F statistic is a measure of instrument strength that is related to the proportion of variance in the phenotype explained by the genetic variants (R^2^), sample size (N) and the number of instruments (k) by the formula F=R^2^ (N−k−1)/k(1−R^2^) (ref.). The Ri^2^ for instrument i is calculated using the approximation Ri^2^=2×EAF_i_×(1−EAF_i_)×βi^2^, where EAF_i_ is the effect allele frequency, and β_i_ is the estimated genetic effect on exposure . An F statistic of ≥10 indicates a relatively low risk of weak instrument bias in MR analysis. We estimated the power of our study according to the method described by Burgess. Briefly, it calculates power by accounting for GWAS sample size, the proportion of cases in case–control GWASs and the variance explained by genetic instruments for the exposure.

**BOLT-LMM**

We used BOLT-LMM, a software package widely used to deal UK Biobank data, to calibrate the effect. While the mathematical derivations underlying BOLT-LMM are based on a quantitative trait model, BOLT-LMM is also applied to analyze binary traits (simply by treating the binary trait as a quantitative trait). Because BOLT-LMM still uses a linear model (rather than a logistic model) when analyzing binary traits, a transformation is used to convert SNP effect size estimates (“betas”) on the quantitative scale to traditional odds ratios. A reasonable approximation is: log OR = β / (μ * (1 - μ)), where μ = case fraction. Standard errors of SNP effect size estimates were divided by (μ * (1 - μ)) when applying the above transformation to obtain log odds ratios. A more sophisticated transformation is described here: http://cnsgenomics.com/shiny/LMOR/.

**Table S4. GWAS summary statistics: source and description**

| **Phenotypes** | **Consortium** | **Sample size (cases)** | **Download** |
| --- | --- | --- | --- |
| Glucosamine supplement | UKB WGS consortium | 360016 (75603) | https://broad-ukb-sumstats-us-east-1.s3.amazonaws.com/round2/additive-tsvs/6179_2.gwas.imputed_v3.both_sexes.tsv.bgz |
| Chondroitin product | UKB WGS consortium | 361141 (6498) | https://broad-ukb-sumstats-us-east-1.s3.amazonaws.com/round2/additive-tsvs/20003_1187.gwas.imputed_v3.both_sexes.tsv.bgz |
| Vitamin supplement | UKB WGS consortium | 54162 (23289) | https://broad-ukb-sumstats-us-east-1.s3.amazonaws.com/round2/additive-tsvs/104670.gwas.imputed_v3.both_sexes.tsv.bgz |
| Osteoarthritis | UKB WGS consortium | 462933  (38472) | https://broad-ukb-sumstats-us-east-1.s3.amazonaws.com/round2/additive-tsvs/20002_1465.gwas.imputed_v3.both_sexes.tsv.bgz |
| All-cause dementia | NA | 172517 (5933) | <https://gwas.mrcieu.ac.uk/datasets/finn-b-KRA_PSY_DEMENTIA_EXMORE/> |
| Alzheimer’s disease | IGAP | 54162 (17008) | <https://gwas.mrcieu.ac.uk/datasets/ieu-a-297/> |
| Vascular dementia | NA | 212389 (881) | <https://gwas.mrcieu.ac.uk/datasets/finn-b-F5_VASCDEM/> |

UKB, UK Biobank; WGS, whole-genome sequencing; IGAP, International Genomics of Alzheimer's Project.

**Table S5. Summary information on glucosamine SNPs used as genetic instruments for the Mendelian randomization analyses**

| **SNP** | **CHR** | **POS** | **A1** | **A2** | **Beta** | **SE** | **P-value** | **F-statistics** |
| --- | --- | --- | --- | --- | --- | --- | --- | --- |
| rs10740101 | 10 | 64746142 | G | A | 0.127 | 0.001 | 4.42E-08 | 56 |
| rs10840899 | 12 | 17996887 | A | G | -0.108 | 0.001 | 4.75E-08 | 43 |
| rs1509952 | 10 | 64739668 | T | C | 0.127 | 0.001 | 3.96E-08 | 55 |
| rs4746746 | 10 | 64699981 | T | C | 0.126 | 0.001 | 4.81E-08 | 58 |
| rs6479846 | 10 | 64655471 | C | A | 0.126 | 0.001 | 4.87E-08 | 55 |
| rs6479848 | 10 | 64659101 | C | T | 0.126 | 0.001 | 4.94E-08 | 52 |
| rs6479860 | 10 | 64745865 | T | C | 0.127 | 0.001 | 4.58E-08 | 56 |
| rs7100204 | 10 | 64640543 | C | T | 0.127 | 0.001 | 4.02E-08 | 50 |
| rs7100320 | 10 | 64640594 | C | T | 0.129 | 0.001 | 2.91E-08 | 58 |
| rs7665570^a^ | 4 | 42152351 | A | C | 0.123 | 0.001 | 4.34E-08 | 46 |

^a^ SNPs associated with potential confounders. SNP: single nucleotide polymorphism - genetic instrument; CHR: chromosome; POS: position; A1: effect allele; A2: other allele; SE: standard error.

**Table S6. Summary information on chondroitin SNPs used as genetic instruments for the Mendelian randomization analyses**

| **SNP** | **CHR** | **POS** | **A1** | **A2** | **Beta** | **SE** | **P-value** |
| --- | --- | --- | --- | --- | --- | --- | --- |
| rs10238899 | 7 | 153492868 | C | T | -0.128 | 0.001 | 2.81E-08 |
| rs10246938^a^ | 7 | 153466423 | T | A | -0.129 | 0.001 | 1.31E-08 |
| rs10247195^a^ | 7 | 153466623 | G | A | -0.129 | 0.001 | 1.34E-08 |
| rs10263816 | 7 | 153470742 | G | A | -0.129 | 0.001 | 1.28E-08 |
| rs10268402^a^ | 7 | 153485627 | C | T | -0.125 | 0.001 | 3.73E-08 |
| rs10282264^a^ | 7 | 153492809 | T | C | -0.125 | 0.001 | 3.97E-08 |
| rs11240229 | 1 | 204576522 | T | C | 0.163 | 0.001 | 5.55E-09 |
| rs11584700 | 1 | 204576983 | G | A | 0.163 | 0.001 | 5.23E-09 |
| rs11588857^a^ | 1 | 204587047 | A | G | 0.164 | 0.001 | 4.16E-09 |
| rs12040459^a^ | 1 | 204564558 | A | G | 0.157 | 0.001 | 2.71E-08 |
| rs12040520^a^ | 1 | 204564762 | A | G | 0.153 | 0.001 | 4.31E-08 |
| rs12044599^a^ | 1 | 204564714 | G | A | 0.153 | 0.001 | 4.18E-08 |
| rs12046747 | 1 | 204593696 | A | G | 0.154 | 0.001 | 2.02E-08 |
| rs16853666 | 1 | 204403856 | C | T | 0.154 | 0.001 | 3.30E-08 |
| rs16854023 | 1 | 204551830 | C | T | 0.170 | 0.001 | 5.47E-09 |
| rs2098112^a^ | 7 | 153487944 | A | G | -0.124 | 0.001 | 4.19E-08 |
| rs2159462 | 7 | 153478989 | A | G | -0.127 | 0.001 | 2.49E-08 |
| rs2533123^a^ | 7 | 153483021 | G | A | -0.125 | 0.001 | 3.61E-08 |
| rs2533125^a^ | 7 | 153473188 | G | A | -0.128 | 0.001 | 1.81E-08 |
| rs2533126^a^ | 7 | 153473086 | A | G | -0.128 | 0.001 | 1.81E-08 |
| rs2533128^a^ | 7 | 153471543 | G | A | -0.127 | 0.001 | 2.07E-08 |
| rs2533133 | 7 | 153466068 | A | G | -0.129 | 0.001 | 1.47E-08 |
| rs2533137 | 7 | 153465680 | C | G | -0.128 | 0.001 | 1.74E-08 |
| rs2533145^a^ | 7 | 153463438 | G | T | -0.129 | 0.001 | 1.29E-08 |
| rs2533148^a^ | 7 | 153462806 | C | T | -0.130 | 0.001 | 1.11E-08 |
| rs2533196 | 7 | 153489530 | A | G | -0.128 | 0.001 | 2.85E-08 |
| rs2533273^a^ | 7 | 153485282 | A | C | -0.131 | 0.001 | 9.57E-09 |
| rs2538462 | 7 | 153497853 | C | T | -0.137 | 0.001 | 3.73E-08 |
| rs2622103 | 7 | 153496171 | A | G | -0.138 | 0.001 | 1.28E-08 |
| rs2622166^a^ | 7 | 153486905 | A | G | -0.125 | 0.001 | 4.10E-08 |
| rs2622170^a^ | 7 | 153486033 | A | T | -0.125 | 0.001 | 3.83E-08 |
| rs2622171^a^ | 7 | 153485893 | G | A | -0.126 | 0.001 | 3.20E-08 |
| rs2622185 | 7 | 153472469 | G | A | -0.129 | 0.001 | 1.33E-08 |
| rs2622217^a^ | 7 | 153464429 | C | G | -0.127 | 0.001 | 2.05E-08 |
| rs2622225^a^ | 7 | 153492123 | G | A | -0.125 | 0.001 | 4.02E-08 |
| rs2622226^a^ | 7 | 153491516 | G | A | -0.125 | 0.001 | 3.55E-08 |
| rs2907674^a^ | 7 | 153471050 | G | A | -0.128 | 0.001 | 1.71E-08 |
| rs3747630 | 1 | 204588140 | A | G | 0.156 | 0.001 | 4.53E-09 |
| rs3747631^a^ | 1 | 204587569 | C | G | 0.163 | 0.001 | 5.01E-09 |
| rs3789044 | 1 | 204589101 | A | G | 0.154 | 0.001 | 2.09E-08 |
| rs55678522 | 1 | 204573481 | A | G | 0.163 | 0.001 | 5.48E-09 |
| rs55979051^a^ | 1 | 204567041 | G | A | 0.156 | 0.001 | 3.33E-08 |
| rs59883612 | 7 | 153484125 | C | T | -0.127 | 0.001 | 3.99E-08 |
| rs60009449 | 7 | 153489996 | C | T | -0.129 | 0.001 | 3.32E-08 |
| rs61817482^a^ | 1 | 204570347 | A | G | 0.157 | 0.001 | 2.63E-08 |
| rs6958769^a^ | 7 | 153487814 | T | C | -0.125 | 0.001 | 4.14E-08 |
| rs73488191^a^ | 7 | 153465730 | G | A | -0.129 | 0.001 | 1.43E-08 |

^a^ SNPs associated with potential confounders. SNP: single nucleotide polymorphism - genetic instrument; CHR: chromosome; POS: position; A1: effect allele; A2: other allele; SE: standard error.

**Table S7. Summary information on vitamin supplement SNPs used as genetic instruments for the Mendelian randomization analyses**

| **SNP** | **CHR** | **POS** | **A1** | **A2** | **Beta** | **SE** | **P-value** |
| --- | --- | --- | --- | --- | --- | --- | --- |
| rs10005662 | 4 | 42178259 | T | C | -0.042 | 0.004 | 2.84E-06 |
| rs10010204 | 4 | 42162182 | C | G | -0.042 | 0.004 | 2.31E-06 |
| rs10034294 | 4 | 42187640 | T | C | -0.051 | 0.005 | 5.80E-07 |
| rs10459544 | 14 | 74419191 | G | C | -0.045 | 0.005 | 3.72E-06 |
| rs11159045 | 14 | 74378984 | T | C | -0.045 | 0.005 | 4.85E-06 |
| rs11559994 | 12 | 40336494 | A | G | -0.034 | 0.004 | 4.50E-06 |
| rs12590001 | 14 | 74368920 | C | T | -0.045 | 0.005 | 4.80E-06 |
| rs1963798 | 14 | 74372862 | C | T | -0.045 | 0.005 | 4.79E-06 |
| rs2079632 | 14 | 74421212 | G | C | -0.046 | 0.005 | 3.03E-06 |
| rs28591364 | 4 | 42174012 | G | T | -0.042 | 0.004 | 2.73E-06 |
| rs28668005 | 4 | 42174395 | T | C | -0.042 | 0.004 | 2.99E-06 |
| rs28687702 | 4 | 42181516 | T | G | -0.042 | 0.004 | 3.00E-06 |
| rs28719870^a^ | 4 | 42157823 | G | A | -0.052 | 0.005 | 1.51E-07 |
| rs34902783 | 4 | 42184017 | C | A | -0.042 | 0.004 | 2.84E-06 |
| rs35079923 | 4 | 42184622 | T | A | -0.042 | 0.004 | 2.98E-06 |
| rs4293573 | 2 | 161877629 | T | G | 0.032 | 0.003 | 4.62E-06 |
| rs4299622 | 4 | 42164386 | G | A | -0.042 | 0.004 | 2.55E-06 |
| rs486112 | 10 | 43243510 | G | A | -0.042 | 0.004 | 4.40E-06 |
| rs4903163 | 14 | 74451171 | C | T | -0.046 | 0.005 | 3.17E-06 |
| rs493230 | 10 | 43247120 | A | G | -0.042 | 0.004 | 4.55E-06 |
| rs507485 | 10 | 43250110 | C | A | -0.059 | 0.005 | 3.04E-07 |
| rs538429 | 10 | 43251146 | T | C | -0.042 | 0.004 | 4.86E-06 |
| rs56087460 | 4 | 42171632 | A | G | -0.042 | 0.004 | 2.74E-06 |
| rs618687 | 10 | 43232419 | G | A | -0.042 | 0.004 | 3.98E-06 |
| rs671429 | 10 | 43248258 | C | T | -0.042 | 0.004 | 4.28E-06 |
| rs677749 | 10 | 43243001 | C | A | -0.042 | 0.004 | 3.98E-06 |
| rs6811916 | 4 | 42183856 | C | T | -0.042 | 0.004 | 2.79E-06 |
| rs6828811^a^ | 4 | 42161066 | C | T | -0.052 | 0.005 | 1.23E-07 |
| rs6857720^a^ | 4 | 42158529 | A | T | -0.051 | 0.005 | 2.43E-07 |
| rs7141392 | 14 | 74428286 | G | A | -0.046 | 0.005 | 3.17E-06 |
| rs71608101 | 4 | 42168329 | T | C | -0.042 | 0.004 | 2.57E-06 |
| rs71608102 | 4 | 42168399 | T | G | -0.042 | 0.004 | 2.55E-06 |
| rs71648648 | 1 | 241472176 | A | G | -0.077 | 0.008 | 2.16E-06 |
| rs7665570 | 4 | 42152351 | A | C | -0.042 | 0.004 | 4.22E-06 |
| rs7960023 | 12 | 40329432 | C | A | -0.034 | 0.004 | 3.94E-06 |
| rs8016802 | 14 | 74462213 | G | A | -0.045 | 0.005 | 4.64E-06 |

^a^ SNPs associated with potential confounders. SNP: single nucleotide polymorphism - genetic instrument; CHR: chromosome; POS: position; A1: effect allele; A2: other allele; SE: standard error.

**Table S8.** **Summary information on osteoarthritis SNPs used as genetic instruments for the Mendelian randomization analyses**

| **SNP** | **CHR** | **POS** | **A1** | **A2** | **Beta** | **SE** | **P-value** |
| --- | --- | --- | --- | --- | --- | --- | --- |
| rs10218792 | 1 | 245750932 | G | T | 0.041 | 0.03 | 2.03E-08 |
| rs2061027 | 2 | 33434336 | A | G | 0.041 | 0.02 | 3.21E-13 |
| rs62262139 | 3 | 50022049 | A | G | 0.043 | 0.02 | 9.16E-11 |
| rs12154055 | 6 | 44449697 | G | A | 0.032 | 0.02 | 2.73E-08 |
| rs919642 | 9 | 116911147 | T | A | 0.054 | 0.03 | 8.69E-15 |
| rs11031191 | 11 | 30774280 | T | G | 0.036 | 0.02 | 1.42E-08 |
| rs1149620 | 11 | 76506572 | T | A | 0.043 | 0.03 | 6.93E-10 |
| rs317630 | 12 | 69637847 | T | C | 0.041 | 0.03 | 2.05E-08 |
| rs2171126 | 12 | 94167220 | T | C | 0.033 | 0.02 | 9.16E-10 |
| rs75621460 | 19 | 41833784 | A | G | 0.152 | 0.09 | 1.61E-15 |
| rs3771501 | 2 | 70717653 | A | G | 0.055 | 0.03 | 4.20E-16 |

SNP: single nucleotide polymorphism - genetic instrument; CHR: chromosome; POS: position; A1: effect allele; A2: other allele; SE: standard error.

**Table S9. Potential confounders of exposures SNPs under the condition of P < 5×10^-8^ in the PhenoScanner database**

| **Exposure** | **Excluded SNP** | **Trait** |
| --- | --- | --- |
| Glucosamine | rs7665570 | Allergic disease |
|  |  | Average weekly red wine intake |
|  |  | Cause of death: large cell |
|  |  | Frequency of tiredness or lethargy in last 2 weeks |
|  |  | Getting up in morning |
|  |  | Hayfever, allergic rhinitis or eczema |
|  |  | Irritability |
|  |  | Mood swings |
|  |  | Neuroticism score |
|  |  | Pack years adult smoking as proportion of life span exposed to smoking |
|  |  | Self-reported fracture hand |
|  |  | Worrier or anxious feelings |
|  |  | Worry too long after embarrassment |
| Chondroitin | rs10246938 | Average weekly beer plus cider intake |
|  |  | Smoking status: current |
|  | rs10247195 | Average weekly beer plus cider intake |
|  |  | Smoking status: current |
|  | rs10268402 | Average weekly beer plus cider intake |
|  |  | Current tobacco smoking |
|  |  | Smoking status: current |
|  | rs10282264 | Average weekly beer plus cider intake |
|  |  | Current tobacco smoking |
|  |  | Smoking status: current |
|  | rs11588857 | College completion |
|  |  | Years of education |
|  |  | Cognitive ability multi trait analysis |
|  |  | Educational attainment years of education |
|  |  | Intelligence multi trait analysis |
|  |  | Alcohol usually taken with meals |
|  |  | Average weekly beer plus cider intake |
|  |  | Forced expiratory volume in 1-second, best measure |
|  |  | Qualifications: college or university degree |
|  |  | Qualifications: none |
|  |  | Qualifications: other professional qualifications |
|  |  | Sodium in urine |
|  |  | College completion |
|  |  | Years of educational attainment in females |
|  |  | Years of educational attainment |
|  |  | Years of educational attainment |
|  | rs12040459 | Average weekly red wine intake |
|  |  | Forced expiratory volume in 1-second, best measure |
|  |  | Forced vital capacity, best measure |
|  |  | Qualifications: college or university degree |
|  |  | Qualifications: other professional qualifications |
|  |  | Sodium in urine |
|  |  | Years of educational attainment in females |
|  |  | Years of educational attainment |
|  | rs12040520 | Average weekly red wine intake |
|  |  | Forced expiratory volume in 1-second, best measure |
|  |  | Forced vital capacity, best measure |
|  |  | Qualifications: college or university degree |
|  |  | Qualifications: other professional qualifications |
|  |  | Sodium in urine |
|  |  | Years of educational attainment in females |
|  |  | Years of educational attainment |
|  | rs12044599 | Average weekly red wine intake |
|  |  | Forced expiratory volume in 1-second, best measure |
|  |  | Forced vital capacity, best measure |
|  |  | Qualifications: college or university degree |
|  |  | Qualifications: other professional qualifications |
|  |  | Sodium in urine |
|  |  | Years of educational attainment in females |
|  |  | Years of educational attainment |
|  | rs2098112 | Crohns disease |
|  |  | Ulcerative colitis |
|  |  | Average weekly beer plus cider intake |
|  |  | Current tobacco smoking |
|  |  | Smoking status: current |
|  | rs2533123 | Average weekly beer plus cider intake |
|  |  | Smoking status: current |
|  | rs2533125 | Average weekly beer plus cider intake |
|  |  | Smoking status: current |
|  | rs2533126 | Average weekly beer plus cider intake |
|  |  | Smoking status: current |
|  | rs2533128 | Average weekly beer plus cider intake |
|  |  | Smoking status: current |
|  | rs2533145 | Average weekly beer plus cider intake |
|  |  | Smoking status: current |
|  | rs2533148 | Crohns disease |
|  |  | Average weekly beer plus cider intake |
|  |  | Smoking status: current |
|  | rs2533273 | Average weekly beer plus cider intake |
|  |  | Current tobacco smoking |
|  |  | Smoking status: current |
|  | rs2622166 | Average weekly beer plus cider intake |
|  |  | Current tobacco smoking |
|  |  | Smoking status: current |
|  | rs2622170 | Average weekly beer plus cider intake |
|  |  | Current tobacco smoking |
|  |  | Smoking status: current |
|  | rs2622171 | Average weekly beer plus cider intake |
|  |  | Current tobacco smoking |
|  |  | Smoking status: current |
|  | rs2622217 | Average weekly beer plus cider intake |
|  |  | Smoking status: current |
|  | rs2622225 | Average weekly beer plus cider intake |
|  |  | Current tobacco smoking |
|  |  | Smoking status: current |
|  | rs2622226 | Average weekly beer plus cider intake |
|  |  | Current tobacco smoking |
|  |  | Smoking status: current |
|  | rs2907674 | Average weekly beer plus cider intake |
|  |  | Smoking status: current |
|  | rs3747631 | Alcohol usually taken with meals |
|  |  | Average weekly beer plus cider intake |
|  |  | Forced expiratory volume in 1-second, best measure |
|  |  | Qualifications: college or university degree |
|  |  | Qualifications: none |
|  |  | Qualifications: other professional qualifications |
|  |  | Sodium in urine |
|  |  | Years of educational attainment in females |
|  |  | Years of educational attainment |
|  | rs55979051 | Average weekly red wine intake |
|  |  | Forced expiratory volume in 1-second, best measure |
|  |  | Forced vital capacity, best measure |
|  |  | Qualifications: college or university degree |
|  |  | Qualifications: other professional qualifications |
|  |  | Sodium in urine |
|  |  | Years of educational attainment in females |
|  |  | Years of educational attainment |
|  | rs61817482 | Average weekly red wine intake |
|  |  | Forced expiratory volume in 1-second, best measure |
|  |  | Forced vital capacity, best measure |
|  |  | Qualifications: college or university degree |
|  |  | Qualifications: other professional qualifications |
|  |  | Sodium in urine |
|  |  | Years of educational attainment in females |
|  |  | Years of educational attainment |
|  | rs6958769 | Average weekly beer plus cider intake |
|  |  | Current tobacco smoking |
|  |  | Smoking status: current |
|  | rs73488191 | Average weekly beer plus cider intake |
|  |  | Smoking status: current |
| Vitamin supplement | rs28719870 | Average weekly red wine intake |
|  |  | Cause of death: large cell |
|  |  | Irritability |
|  |  | Neuroticism score |
|  |  | Pack years adult smoking as proportion of life span exposed to smoking |
|  |  | Seen doctor for nerves, anxiety, tension or depression |
|  |  | Treatment with hydroxocobalamin |
|  |  | Worrier or anxious feelings |
|  | rs6828811 | Average weekly red wine intake |
|  |  | Cause of death: large cell |
|  |  | Irritability |
|  |  | Miserableness |
|  |  | Neuroticism score |
|  |  | Seen doctor for nerves, anxiety, tension or depression |
|  |  | Treatment with hydroxocobalamin |
|  |  | Worrier or anxious feelings |
|  | rs6857720 | Average weekly red wine intake |
|  |  | Cause of death: large cell |
|  |  | Irritability |
|  |  | Job involves shift work |
|  |  | Neuroticism score |
|  |  | Pack years adult smoking as proportion of life span exposed to smoking |
|  |  | Seen doctor for nerves, anxiety, tension or depression |
|  |  | Treatment with hydroxocobalamin |
|  |  | Worrier or anxious feelings |

SNP: single nucleotide polymorphism. All statistical tests were two-sided. A P-value < 5 × 10^−8^ with a potential confounder in the PhenoScanner database was considered genome-wide significant and removed.

**Table S10. Summary information on all-cause dementia for the 9 genome-wide significant SNPs associated with glucosamine.**

| **SNP** | **CHR** | **POS** | **A1** | **A2** | **Beta** | **SE** | **P-value** |
| --- | --- | --- | --- | --- | --- | --- | --- |
| rs10740101 | 10 | 64746142 | G | A | -0.024 | 0.023 | 0.285 |
| rs10840899 | 12 | 17996887 | A | G | 0.001 | 0.022 | 0.979 |
| rs1509952 | 10 | 64739668 | T | C | -0.024 | 0.023 | 0.291 |
| rs4746746 | 10 | 64699981 | T | C | -0.023 | 0.023 | 0.306 |
| rs6479846 | 10 | 64655471 | C | A | -0.023 | 0.023 | 0.314 |
| rs6479848 | 10 | 64659101 | C | T | -0.023 | 0.023 | 0.310 |
| rs6479860 | 10 | 64745865 | T | C | -0.025 | 0.023 | 0.283 |
| rs7100204 | 10 | 64640543 | C | T | -0.021 | 0.023 | 0.372 |
| rs7100320 | 10 | 64640594 | C | T | -0.020 | 0.023 | 0.379 |

SNP: single nucleotide polymorphism - genetic instrument; CHR: chromosome; POS: position; A1: effect allele; A2: other allele; SE: standard error.

**Table S11. Summary information on Alzheimer’ disease for the 9 genome-wide significant SNPs associated with glucosamine.**

| **SNP** | **CHR** | **POS** | **A1** | **A2** | **Beta** | **SE** | **P-value** |
| --- | --- | --- | --- | --- | --- | --- | --- |
| rs10740101 | 10 | 64746142 | G | A | -0.022 | 0.018 | 0.234 |
| rs10840899 | 12 | 17996887 | A | G | 0.009 | 0.016 | 0.572 |
| rs1509952 | 10 | 64739668 | T | C | -0.020 | 0.018 | 0.269 |
| rs4746746 | 10 | 64699981 | T | C | -0.018 | 0.019 | 0.329 |
| rs6479846 | 10 | 64655471 | C | A | -0.019 | 0.018 | 0.301 |
| rs6479848 | 10 | 64659101 | C | T | -0.021 | 0.018 | 0.242 |
| rs6479860 | 10 | 64745865 | T | C | -0.020 | 0.019 | 0.290 |
| rs7100204 | 10 | 64640543 | C | T | -0.026 | 0.019 | 0.161 |
| rs7100320 | 10 | 64640594 | C | T | -0.026 | 0.019 | 0.161 |

SNP: single nucleotide polymorphism - genetic instrument; CHR: chromosome; POS: position; A1: effect allele; A2: other allele; SE: standard error.

**Table S12. Summary information on vascular dementia for the 9 genome-wide significant SNPs associated with glucosamine.**

| **SNP** | **CHR** | **POS** | **A1** | **A2** | **Beta** | **SE** | **P-value** |
| --- | --- | --- | --- | --- | --- | --- | --- |
| rs10740101 | 10 | 64746142 | G | A | -0.104 | 0.053 | 0.053 |
| rs10840899 | 12 | 17996887 | A | G | -0.049 | 0.051 | 0.336 |
| rs1509952 | 10 | 64739668 | T | C | -0.092 | 0.053 | 0.084 |
| rs4746746 | 10 | 64699981 | T | C | -0.103 | 0.053 | 0.055 |
| rs6479846 | 10 | 64655471 | C | A | -0.109 | 0.054 | 0.041 |
| rs6479848 | 10 | 64659101 | C | T | -0.111 | 0.054 | 0.037 |
| rs6479860 | 10 | 64745865 | T | C | -0.104 | 0.053 | 0.052 |
| rs7100204 | 10 | 64640543 | C | T | -0.109 | 0.054 | 0.043 |
| rs7100320 | 10 | 64640594 | C | T | -0.108 | 0.054 | 0.044 |

SNP: single nucleotide polymorphism - genetic instrument; CHR: chromosome; POS: position; A1: effect allele; A2: other allele; SE: standard error.

**Table S13. Results of MR Steiger direction test for glucosamine on dementia.**

| **Exposures** | **Outcome** | **r^2^ exposure** | **r^2^ outcome** | **Correct causal direction** | **Steiger P-value** |
| --- | --- | --- | --- | --- | --- |
| Glucosamine | All cause dementia | 0.1404263 | 0.004215 | TRUE | 4.53×10^-9^ |
|  | Alzheimer’s disease | 0.1404263 | 0.003833 | TRUE | 5.02×10^-9^ |
|  | Vascular dementia | 0.1404263 | 0.003125 | TRUE | 3.85×10^-9^ |

**Table S14. Independent instruments used for multivariable MR**

| **SNP** | **A1** | **A2** | **Exposure** | | | | | | | | | | | |
| --- | --- | --- | --- | --- | --- | --- | --- | --- | --- | --- | --- | --- | --- | --- |
|  |  |  | **Glucosamine** | | | **Chondroitin** | | | **Vitamin supplement** | | | **Osteoarthritis** | | |
|  |  |  | **Beta** | **SE** | **P-value** | **Beta** | **SE** | **P-value** | **Beta** | **SE** | **P-value** | **Beta** | **SE** | **P-value** |
| rs10238899 | C | T | -0.001 | 0.001 | 5.48E-01 | -0.128 | 0.001 | 2.81E-08 | -0.006 | 0.003 | 3.59E-02 | -0.005 | 0.003 | 4.36E-02 |
| rs10263816 | G | A | -0.001 | 0.001 | 3.90E-01 | -0.129 | 0.001 | 1.28E-08 | -0.005 | 0.003 | 8.30E-02 | 0.005 | 0.003 | 9.22E-02 |
| rs11240229 | T | C | 0.002 | 0.001 | 3.11E-02 | 0.163 | 0.001 | 5.55E-09 | 0.007 | 0.004 | 7.35E-02 | -0.005 | 0.003 | 3.68E-02 |
| rs2533137 | C | G | -0.001 | 0.001 | 4.06E-01 | -0.128 | 0.001 | 1.74E-08 | -0.005 | 0.003 | 8.03E-02 | 0.006 | 0.003 | 5.77E-02 |
| rs11584700 | G | A | 0.002 | 0.001 | 3.15E-02 | 0.163 | 0.001 | 5.23E-09 | 0.007 | 0.004 | 7.41E-02 | -0.007 | 0.004 | 2.67E-02 |
| rs12046747 | A | G | 0.003 | 0.001 | 1.39E-02 | 0.154 | 0.001 | 2.02E-08 | 0.007 | 0.004 | 6.13E-02 | -0.003 | 0.004 | 4.33E-02 |
| rs16853666 | C | T | 0.002 | 0.001 | 9.71E-02 | 0.154 | 0.001 | 3.30E-08 | 0.007 | 0.004 | 4.32E-02 | 0.005 | 0.004 | 7.66E-02 |
| rs16854023 | C | T | 0.002 | 0.001 | 5.76E-02 | 0.170 | 0.001 | 5.47E-09 | 0.007 | 0.004 | 6.91E-02 | 0.003 | 0.004 | 1.69E-02 |
| rs2159462 | A | G | -0.001 | 0.001 | 3.89E-01 | -0.127 | 0.001 | 2.49E-08 | -0.005 | 0.003 | 1.03E-01 | 0.004 | 0.003 | 1.57E-02 |
| rs2533133 | A | G | -0.001 | 0.001 | 3.95E-01 | -0.129 | 0.001 | 1.47E-08 | -0.005 | 0.003 | 8.03E-02 | 0.005 | 0.003 | 8.08E-02 |
| rs2533196 | A | G | -0.001 | 0.001 | 5.71E-01 | -0.128 | 0.001 | 2.85E-08 | -0.006 | 0.003 | 5.58E-02 | -0.005 | 0.003 | 6.47E-02 |
| rs2538462 | C | T | -0.001 | 0.001 | 5.84E-01 | -0.137 | 0.001 | 3.73E-08 | -0.007 | 0.003 | 2.42E-02 | -0.005 | 0.003 | 4.67E-02 |
| rs2622103 | A | G | -0.001 | 0.001 | 5.97E-01 | -0.138 | 0.001 | 1.28E-08 | -0.007 | 0.003 | 2.63E-02 | 0.006 | 0.003 | 7.32E-02 |
| rs2622185 | G | A | -0.001 | 0.001 | 3.37E-01 | -0.129 | 0.001 | 1.33E-08 | -0.005 | 0.003 | 8.51E-02 | 0.005 | 0.002 | 8.72E-02 |
| rs3747630 | A | G | 0.002 | 0.001 | 3.32E-02 | 0.156 | 0.001 | 4.53E-09 | 0.006 | 0.004 | 6.89E-02 | -0.007 | 0.004 | 4.62E-02 |
| rs3789044 | A | G | 0.003 | 0.001 | 1.37E-02 | 0.154 | 0.001 | 2.09E-08 | 0.007 | 0.004 | 6.33E-02 | -0.005 | 0.003 | 9.58E-02 |
| rs55678522 | A | G | 0.002 | 0.001 | 3.12E-02 | 0.163 | 0.001 | 5.48E-09 | 0.007 | 0.004 | 7.37E-02 | 0.003 | 0.002 | 4.77E-02 |
| rs59883612 | C | T | -0.001 | 0.001 | 3.94E-01 | -0.127 | 0.001 | 3.99E-08 | -0.005 | 0.003 | 8.40E-02 | 0.003 | 0.001 | 7.45E-02 |
| rs60009449 | C | T | -0.001 | 0.001 | 5.81E-01 | -0.129 | 0.001 | 3.32E-08 | -0.005 | 0.003 | 9.06E-02 | 0.003 | 0.001 | 1.68E-02 |
| rs10740101 | G | A | 0.127 | 0.001 | 4.42E-08 | 0.002 | 0.001 | 6.00E-02 | 0.002 | 0.004 | 6.54E-01 | 0.002 | 0.001 | 2.93E-01 |
| rs10840899 | A | G | -0.108 | 0.001 | 4.75E-08 | -0.003 | 0.001 | 7.86E-03 | -0.003 | 0.003 | 3.61E-01 | 0.003 | 0.001 | 3.21E-02 |
| rs1509952 | T | C | 0.127 | 0.001 | 3.96E-08 | 0.002 | 0.001 | 7.08E-02 | 0.001 | 0.004 | 7.04E-01 | 0.002 | 0.001 | 3.01E-01 |
| rs4746746 | T | C | 0.126 | 0.001 | 4.81E-08 | 0.002 | 0.001 | 8.61E-02 | 0.001 | 0.004 | 7.10E-01 | 0.002 | 0.001 | 5.12E-02 |
| rs6479846 | C | A | 0.126 | 0.001 | 4.87E-08 | 0.002 | 0.001 | 9.26E-02 | 0.000 | 0.004 | 9.63E-01 | 0.003 | 0.001 | 2.12E-02 |
| rs6479848 | C | T | 0.126 | 0.001 | 4.94E-08 | 0.002 | 0.001 | 9.48E-02 | 0.000 | 0.004 | 9.73E-01 | 0.002 | 0.001 | 3.73E-02 |
| rs6479860 | T | C | 0.127 | 0.001 | 4.58E-08 | 0.002 | 0.001 | 6.08E-02 | 0.002 | 0.004 | 6.57E-01 | 0.003 | 0.001 | 4.72E-02 |
| rs7100204 | C | T | 0.127 | 0.001 | 4.02E-08 | 0.002 | 0.001 | 1.13E-01 | 0.001 | 0.004 | 7.91E-01 | 0.002 | 0.001 | 5.89E-01 |
| rs7100320 | C | T | 0.129 | 0.001 | 2.91E-08 | 0.002 | 0.001 | 1.09E-01 | 0.001 | 0.004 | 7.72E-01 | 0.002 | 0.001 | 4.92E-01 |
| rs10005662 | T | C | -0.005 | 0.001 | 3.60E-04 | -0.006 | 0.002 | 4.56E-05 | -0.419 | 0.004 | 2.84E-06 | -0.001 | 0.001 | 5.48E-01 |
| rs10034294 | T | C | -0.005 | 0.001 | 5.82E-04 | -0.008 | 0.002 | 9.43E-06 | -0.508 | 0.005 | 5.80E-07 | -0.001 | 0.001 | 3.90E-01 |
| rs11159045 | T | C | -0.003 | 0.001 | 7.90E-02 | -0.005 | 0.002 | 4.34E-03 | -0.447 | 0.005 | 4.85E-06 | 0.002 | 0.001 | 3.11E-02 |
| rs11559994 | A | G | 0.000 | 0.001 | 9.06E-01 | -0.003 | 0.001 | 8.54E-03 | -0.342 | 0.004 | 4.50E-06 | -0.001 | 0.001 | 4.06E-01 |
| rs12590001 | C | T | -0.002 | 0.001 | 9.21E-02 | -0.005 | 0.002 | 2.75E-03 | -0.448 | 0.005 | 4.80E-06 | 0.002 | 0.001 | 3.15E-02 |
| rs1963798 | C | T | -0.003 | 0.001 | 7.79E-02 | -0.005 | 0.002 | 4.68E-03 | -0.447 | 0.005 | 4.79E-06 | 0.003 | 0.001 | 1.39E-02 |
| rs28591364 | G | T | -0.005 | 0.001 | 3.97E-04 | -0.006 | 0.002 | 3.75E-05 | -0.419 | 0.004 | 2.73E-06 | 0.002 | 0.001 | 9.71E-02 |
| rs28668005 | T | C | -0.005 | 0.001 | 3.59E-04 | -0.006 | 0.002 | 4.40E-05 | -0.418 | 0.004 | 2.99E-06 | 0.002 | 0.001 | 5.76E-02 |
| rs28687702 | T | G | -0.005 | 0.001 | 3.72E-04 | -0.006 | 0.002 | 4.65E-05 | -0.418 | 0.004 | 3.00E-06 | -0.001 | 0.001 | 3.89E-01 |
| rs34902783 | C | A | -0.005 | 0.001 | 3.89E-04 | -0.006 | 0.002 | 4.33E-05 | -0.419 | 0.004 | 2.84E-06 | -0.001 | 0.001 | 3.95E-01 |
| rs4293573 | T | G | 0.000 | 0.001 | 6.32E-01 | 0.003 | 0.001 | 1.27E-02 | 0.322 | 0.003 | 4.62E-06 | -0.001 | 0.001 | 5.71E-01 |
| rs4299622 | G | A | -0.005 | 0.001 | 2.45E-04 | -0.006 | 0.002 | 4.97E-05 | -0.421 | 0.004 | 2.55E-06 | -0.001 | 0.001 | 5.84E-01 |
| rs486112 | G | A | -0.001 | 0.001 | 4.95E-01 | -0.003 | 0.002 | 8.18E-02 | -0.421 | 0.004 | 4.40E-06 | -0.001 | 0.001 | 6.97E-01 |
| rs4903163 | C | T | -0.002 | 0.001 | 1.06E-01 | -0.005 | 0.002 | 3.21E-03 | -0.457 | 0.005 | 3.17E-06 | -0.001 | 0.001 | 2.37E-01 |
| rs493230 | A | G | -0.001 | 0.001 | 5.07E-01 | -0.003 | 0.002 | 8.35E-02 | -0.420 | 0.004 | 4.55E-06 | 0.002 | 0.001 | 4.32E-02 |
| rs507485 | C | A | -0.001 | 0.002 | 4.81E-01 | -0.004 | 0.002 | 6.70E-02 | -0.592 | 0.005 | 3.04E-07 | 0.003 | 0.001 | 3.37E-02 |
| rs538429 | T | C | -0.001 | 0.001 | 4.96E-01 | -0.003 | 0.002 | 9.59E-02 | -0.419 | 0.004 | 4.86E-06 | 0.002 | 0.001 | 2.12E-02 |
| rs56087460 | A | G | -0.005 | 0.001 | 3.75E-04 | -0.006 | 0.002 | 5.87E-05 | -0.420 | 0.004 | 2.74E-06 | -0.001 | 0.001 | 1.94E-01 |
| rs618687 | G | A | -0.001 | 0.001 | 4.86E-01 | -0.003 | 0.002 | 8.95E-02 | -0.422 | 0.004 | 3.98E-06 | -0.001 | 0.001 | 2.89E-01 |
| rs671429 | C | T | -0.001 | 0.001 | 4.81E-01 | -0.003 | 0.002 | 7.91E-02 | -0.421 | 0.004 | 4.28E-06 | 0.002 | 0.001 | 3.95E-01 |
| rs677749 | C | A | -0.001 | 0.001 | 5.15E-01 | -0.003 | 0.002 | 8.75E-02 | -0.423 | 0.004 | 3.98E-06 | -0.001 | 0.001 | 581E-01 |
| rs6811916 | C | T | -0.005 | 0.001 | 3.91E-04 | -0.006 | 0.002 | 4.34E-05 | -0.419 | 0.004 | 2.79E-06 | -0.001 | 0.001 | 6.84E-01 |
| rs7141392 | G | A | -0.002 | 0.001 | 1.04E-01 | -0.005 | 0.002 | 3.27E-03 | -0.457 | 0.005 | 3.17E-06 | -0.001 | 0.001 | 4.97E-01 |
| rs71608101 | T | C | -0.005 | 0.001 | 2.61E-04 | -0.006 | 0.002 | 5.00E-05 | -0.421 | 0.004 | 2.57E-06 | -0.001 | 0.001 | 3.37E-01 |
| rs71608102 | T | G | -0.005 | 0.001 | 2.53E-04 | -0.006 | 0.002 | 4.68E-05 | -0.421 | 0.004 | 2.55E-06 | -0.001 | 0.001 | 1.32E-02 |
| rs71648648 | A | G | -0.004 | 0.002 | 8.52E-02 | -0.006 | 0.003 | 3.40E-02 | -0.767 | 0.008 | 2.16E-06 | -0.001 | 0.001 | 1.27E-02 |
| rs7665570 | A | C | -0.005 | 0.001 | 6.11E-05 | -0.007 | 0.002 | 9.00E-06 | -0.415 | 0.004 | 4.22E-06 | 0.002 | 0.001 | 3.13E-02 |
| rs7960023 | C | A | 0.000 | 0.001 | 9.32E-01 | -0.003 | 0.001 | 7.21E-03 | -0.344 | 0.004 | 3.94E-06 | 0.003 | 0.001 | 5.97E-01 |
| rs8016802 | G | A | -0.002 | 0.001 | 1.24E-01 | -0.005 | 0.002 | 5.42E-03 | -0.454 | 0.005 | 4.64E-06 | -0.001 | 0.001 | 3.37E-01 |
| rs10010204 | C | G | -0.005 | 0.001 | 2.71E-04 | -0.006 | 0.002 | 4.35E-05 | -0.423 | 0.004 | 2.31E-06 | -0.001 | 0.001 | 3.32E-02 |
| rs2079632 | G | C | -0.002 | 0.001 | 1.05E-01 | -0.005 | 0.002 | 3.36E-03 | -0.458 | 0.005 | 3.03E-06 | 0.002 | 0.001 | 1.37E-02 |
| rs35079923 | T | A | -0.005 | 0.001 | 3.72E-04 | -0.006 | 0.002 | 3.98E-05 | -0.418 | 0.004 | 2.98E-06 | 0.003 | 0.001 | 3.12E-02 |
| rs10459544 | G | C | -0.002 | 0.001 | 1.01E-01 | -0.005 | 0.002 | 4.58E-03 | -0.453 | 0.005 | 3.72E-06 | 0.002 | 0.001 | 3.12E-02 |
| rs10218792 | G | T | 0.001 | 0.001 | 3.42E-01 | -0.003 | 0.002 | 2.14E-03 | 0.006 | 0.004 | 6.42E-02 | 0.041 | 0.031 | 2.03E-08 |
| rs2061027 | A | G | 0.001 | 0.001 | 1.91E-01 | -0.008 | 0.001 | 3.03E-02 | 0.005 | 0.003 | 5.23E-02 | 0.041 | 0.023 | 3.21E-13 |
| rs62262139 | A | G | 0.003 | 0.001 | 4.21E-02 | -0.009 | 0.001 | 6.58E-02 | 0.008 | 0.004 | 3.31E-02 | 0.043 | 0.026 | 9.16E-11 |
| rs12154055 | G | A | 0.002 | 0.002 | 6.06E-01 | -0.003 | 0.002 | 6.72E-03 | -0.004 | 0.003 | 7.61E-02 | 0.032 | 0.022 | 2.73E-08 |
| rs919642 | T | A | 0.002 | 0.001 | 2.15E-02 | -0.007 | 0.001 | 1.63E-02 | -0.005 | 0.003 | 2.13E-01 | 0.054 | 0.033 | 8.69E-15 |
| rs11031191 | T | G | 0.001 | 0.001 | 5.39E-02 | -0.006 | 0.002 | 6.51E-02 | -0.007 | 0.003 | 9.13E-02 | 0.036 | 0.025 | 1.42E-08 |
| rs1149620 | T | A | 0.002 | 0.001 | 9.71E-02 | 0.002 | 0.001 | 7.89E-03 | -0.005 | 0.003 | 2.18E-02 | 0.043 | 0.032 | 6.93E-10 |
| rs317630 | T | C | 0.001 | 0.001 | 2.76E-02 | -0.006 | 0.002 | 9.33E-02 | -0.007 | 0.004 | 3.42E-02 | 0.041 | 0.031 | 2.05E-08 |
| rs2171126 | T | C | 0.002 | 0.002 | 4.89E-01 | -0.003 | 0.002 | 2.27E-02 | -0.004 | 0.003 | 5.63E-02 | 0.033 | 0.028 | 9.16E-10 |
| rs75621460 | A | G | 0.003 | 0.001 | 5.95E-01 | -0.005 | 0.002 | 5.40E-02 | 0.007 | 0.004 | 8.51E-02 | 0.152 | 0.091 | 1.61E-15 |
| rs3771501 | A | G | 0.001 | 0.001 | 8.71E-01 | -0.007 | 0.002 | 4.04E-02 | 0.007 | 0.004 | 7.89E-02 | 0.055 | 0.032 | 4.20E-16 |

SNP: single nucleotide polymorphism; A1: effect allele; A2: other allele; SE: standard error.

**Table S15. Summary information on dementia for the genome-wide significant SNPs associated with multivariable Instruments.**

| **SNP** | **A1** | **A2** | **Outcomes** | | | | | | | | |
| --- | --- | --- | --- | --- | --- | --- | --- | --- | --- | --- | --- |
|  |  |  | **All cause dementia** | | | **Alzheimer’s disease** | | | **Vascular dementia** | | |
|  |  |  | **Beta** | **SE** | **P-value** | **Beta** | **SE** | **P-value** | **Beta** | **SE** | **P-value** |
| rs10238899 | C | T | 0.025 | 0.022 | 0.243 | 0.014 | 0.023 | 0.523 | -0.038 | 0.051 | 0.456 |
| rs10263816 | G | A | 0.023 | 0.022 | 0.283 | 0.038 | 0.018 | 0.031 | 0.000 | 0.050 | 0.993 |
| rs11240229 | T | C | -0.036 | 0.025 | 0.158 | 0.010 | 0.019 | 0.603 | -0.054 | 0.059 | 0.364 |
| rs11584700 | G | A | -0.036 | 0.025 | 0.158 | 0.011 | 0.020 | 0.592 | -0.054 | 0.059 | 0.364 |
| rs12046747 | A | G | -0.036 | 0.025 | 0.149 | 0.012 | 0.019 | 0.523 | -0.064 | 0.059 | 0.278 |
| rs16853666 | C | T | -0.014 | 0.023 | 0.528 | -0.020 | 0.019 | 0.285 | -0.042 | 0.053 | 0.432 |
| rs16854023 | C | T | -0.029 | 0.026 | 0.260 | 0.019 | 0.020 | 0.349 | -0.050 | 0.061 | 0.408 |
| rs2159462 | A | G | 0.025 | 0.021 | 0.252 | 0.033 | 0.021 | 0.117 | -0.008 | 0.050 | 0.867 |
| rs2533133 | A | G | 0.016 | 0.022 | 0.455 | 0.039 | 0.018 | 0.033 | -0.014 | 0.050 | 0.776 |
| rs2533196 | A | G | 0.026 | 0.022 | 0.223 | 0.022 | 0.020 | 0.285 | -0.036 | 0.050 | 0.478 |
| rs2538462 | C | T | NA | NA | NA | 0.018 | 0.027 | 0.505 | NA | NA | NA |
| rs2622103 | A | G | 0.022 | 0.022 | 0.321 | 0.016 | 0.024 | 0.511 | -0.021 | 0.051 | 0.681 |
| rs2622185 | G | A | 0.025 | 0.022 | 0.254 | 0.043 | 0.025 | 0.084 | -0.002 | 0.050 | 0.966 |
| rs3747630 | A | G | -0.018 | 0.024 | 0.457 | 0.012 | 0.019 | 0.506 | -0.081 | 0.057 | 0.159 |
| rs3789044 | A | G | -0.036 | 0.025 | 0.153 | -0.013 | 0.018 | 0.463 | -0.059 | 0.059 | 0.318 |
| rs55678522 | A | G | -0.036 | 0.025 | 0.158 | 0.010 | 0.019 | 0.591 | -0.054 | 0.059 | 0.367 |
| rs59883612 | C | T | 0.018 | 0.022 | 0.415 | 0.026 | 0.026 | 0.323 | 0.033 | 0.052 | 0.519 |
| rs60009449 | C | T | 0.025 | 0.022 | 0.244 | 0.019 | 0.024 | 0.434 | -0.038 | 0.051 | 0.455 |
| rs10740101 | G | A | -0.024 | 0.023 | 0.285 | -0.022 | 0.018 | 0.234 | -0.104 | 0.053 | 0.053 |
| rs10840899 | A | G | 0.001 | 0.022 | 0.979 | 0.009 | 0.016 | 0.572 | -0.049 | 0.051 | 0.336 |
| rs1509952 | T | C | -0.024 | 0.023 | 0.291 | -0.020 | 0.018 | 0.269 | -0.092 | 0.053 | 0.084 |
| rs4746746 | T | C | -0.023 | 0.023 | 0.306 | -0.018 | 0.019 | 0.329 | -0.103 | 0.053 | 0.055 |
| rs6479846 | C | A | -0.023 | 0.023 | 0.314 | -0.019 | 0.018 | 0.301 | -0.109 | 0.054 | 0.041 |
| rs6479848 | C | T | -0.023 | 0.023 | 0.310 | -0.021 | 0.018 | 0.242 | -0.111 | 0.054 | 0.037 |
| rs6479860 | T | C | -0.025 | 0.023 | 0.283 | -0.020 | 0.019 | 0.290 | -0.104 | 0.053 | 0.052 |
| rs7100204 | C | T | -0.021 | 0.023 | 0.372 | -0.026 | 0.019 | 0.161 | -0.109 | 0.054 | 0.043 |
| rs7100320 | C | T | -0.020 | 0.023 | 0.379 | -0.026 | 0.019 | 0.161 | -0.108 | 0.054 | 0.044 |
| rs10005662 | T | C | 0.041 | 0.032 | 0.197 | -0.012 | 0.023 | 0.603 | 0.047 | 0.075 | 0.530 |
| rs10034294 | T | C | 0.039 | 0.034 | 0.258 | -0.004 | 0.028 | 0.880 | 0.020 | 0.080 | 0.804 |
| rs11159045 | T | C | 0.075 | 0.035 | 0.034 | 0.021 | 0.024 | 0.371 | 0.113 | 0.082 | 0.168 |
| rs11559994 | A | G | 0.036 | 0.025 | 0.141 | -0.006 | 0.019 | 0.746 | 0.084 | 0.057 | 0.143 |
| rs12590001 | C | T | 0.075 | 0.035 | 0.033 | 0.022 | 0.023 | 0.336 | 0.113 | 0.082 | 0.167 |
| rs1963798 | C | T | 0.075 | 0.035 | 0.034 | 0.013 | 0.022 | 0.563 | 0.113 | 0.082 | 0.168 |
| rs28591364 | G | T | 0.041 | 0.032 | 0.199 | -0.012 | 0.022 | 0.590 | 0.047 | 0.075 | 0.530 |
| rs28668005 | T | C | 0.041 | 0.032 | 0.201 | -0.012 | 0.023 | 0.601 | 0.049 | 0.075 | 0.511 |
| rs28687702 | T | G | 0.041 | 0.032 | 0.196 | -0.013 | 0.023 | 0.573 | 0.047 | 0.075 | 0.529 |
| rs34902783 | C | A | 0.041 | 0.032 | 0.199 | -0.014 | 0.023 | 0.543 | 0.047 | 0.075 | 0.532 |
| rs4293573 | T | G | 0.056 | 0.023 | 0.014 | 0.004 | 0.017 | 0.823 | 0.083 | 0.053 | 0.118 |
| rs4299622 | G | A | 0.041 | 0.032 | 0.201 | -0.014 | 0.022 | 0.531 | 0.048 | 0.075 | 0.526 |
| rs486112 | G | A | -0.019 | 0.032 | 0.554 | 0.012 | 0.024 | 0.626 | 0.037 | 0.075 | 0.616 |
| rs4903163 | C | T | 0.072 | 0.036 | 0.043 | 0.029 | 0.024 | 0.222 | 0.122 | 0.082 | 0.138 |
| rs493230 | A | G | -0.018 | 0.032 | 0.567 | 0.019 | 0.025 | 0.448 | 0.042 | 0.075 | 0.573 |
| rs507485 | C | A | -0.019 | 0.033 | 0.557 | 0.053 | 0.030 | 0.075 | 0.029 | 0.076 | 0.704 |
| rs538429 | T | C | -0.018 | 0.032 | 0.574 | 0.017 | 0.023 | 0.475 | 0.039 | 0.075 | 0.601 |
| rs56087460 | A | G | 0.040 | 0.032 | 0.209 | -0.012 | 0.022 | 0.601 | 0.040 | 0.075 | 0.594 |
| rs618687 | G | A | -0.019 | 0.032 | 0.550 | 0.016 | 0.024 | 0.499 | 0.033 | 0.075 | 0.655 |
| rs671429 | C | T | -0.019 | 0.032 | 0.558 | 0.017 | 0.023 | 0.480 | 0.038 | 0.075 | 0.611 |
| rs677749 | C | A | -0.019 | 0.032 | 0.554 | 0.013 | 0.024 | 0.595 | 0.037 | 0.075 | 0.617 |
| rs6811916 | C | T | 0.041 | 0.032 | 0.199 | -0.013 | 0.023 | 0.557 | 0.047 | 0.075 | 0.532 |
| rs7141392 | G | A | 0.072 | 0.036 | 0.043 | 0.026 | 0.023 | 0.261 | 0.122 | 0.082 | 0.138 |
| rs71608101 | T | C | 0.040 | 0.032 | 0.207 | -0.012 | 0.022 | 0.598 | 0.040 | 0.075 | 0.592 |
| rs71608102 | T | G | 0.040 | 0.032 | 0.207 | -0.012 | 0.022 | 0.602 | 0.040 | 0.075 | 0.592 |
| rs71648648 | A | G | 0.035 | 0.057 | 0.536 | 0.031 | 0.044 | 0.483 | -0.273 | 0.132 | 0.039 |
| rs7665570 | A | C | 0.046 | 0.030 | 0.126 | 0.007 | 0.023 | 0.762 | 0.063 | 0.070 | 0.364 |
| rs7960023 | C | A | 0.036 | 0.025 | 0.141 | -0.003 | 0.018 | 0.873 | 0.083 | 0.057 | 0.143 |
| rs8016802 | G | A | 0.078 | 0.035 | 0.025 | 0.022 | 0.023 | 0.334 | 0.109 | 0.081 | 0.177 |
| rs10010204 | C | G | 0.042 | 0.032 | 0.194 | -0.0113 ^a^ | 0.0222 | 0.609 | 0.048 | 0.075 | 0.524 |
| rs2079632 | G | C | 0.072 | 0.036 | 0.043 | 0.0244 ^a^ | 0.023 | 0.289 | 0.122 | 0.082 | 0.138 |
| rs35079923 | T | A | 0.041 | 0.032 | 0.199 | -0.0153 ^a^ | 0.0231 | 0.507 | 0.047 | 0.075 | 0.532 |
| rs10459544 | G | C | 0.074 | 0.035 | 0.036 | 0.0241 ^a^ | 0.023 | 0.295 | 0.113 | 0.082 | 0.168 |
| rs2533137 | C | G | NA | NA | NA | 0.0404 ^a^ | 0.018 | 0.022 | NA | NA | NA |
| rs10218792 | G | T | 0.073 | 0.033 | 0.044 | 0.013 | 0.023 | 0.613 | 0.057 | 0.075 | 0.511 |
| rs2061027 | A | G | 0.046 | 0.035 | 0.231 | 0.036 | 0.018 | 0.032 | 0.059 | 0.075 | 0.532 |
| rs62262139 | A | G | 0.065 | 0.035 | 0.041 | 0.011 | 0.019 | 0.613 | 0.057 | 0.075 | 0.516 |
| rs12154055 | G | A | 0.074 | 0.037 | 0.040 | 0.010 | 0.020 | 0.573 | 0.057 | 0.075 | 0.542 |
| rs919642 | T | A | 0.052 | 0.032 | 0.219 | 0.014 | 0.019 | 0.512 | 0.073 | 0.053 | 0.149 |
| rs11031191 | T | G | 0.051 | 0.033 | 0.221 | 0.021 | 0.019 | 0.255 | 0.058 | 0.075 | 0.516 |
| rs1149620 | T | A | 0.042 | 0.034 | 0.176 | 0.017 | 0.010 | 0.447 | 0.045 | 0.075 | 0.616 |
| rs317630 | T | C | 0.041 | 0.033 | 0.189 | 0.031 | 0.021 | 0.128 | 0.131 | 0.082 | 0.138 |
| rs2171126 | T | C | 0.053 | 0.033 | 0.024 | 0.035 | 0.018 | 0.043 | 0.052 | 0.075 | 0.583 |
| rs75621460 | A | G | 0.044 | 0.031 | 0.211 | 0.023 | 0.020 | 0.255 | 0.039 | 0.076 | 0.704 |
| rs3771501 | A | G | 0.049 | 0.031 | 0.564 | 0.017 | 0.027 | 0.602 | 0.047 | 0.075 | 0.602 |

^a^ Removing the SNP for being palindromic with intermediate allele frequencies. SNP: single nucleotide polymorphism; A1: effect allele; A2: other allele; SE: standard error.

**
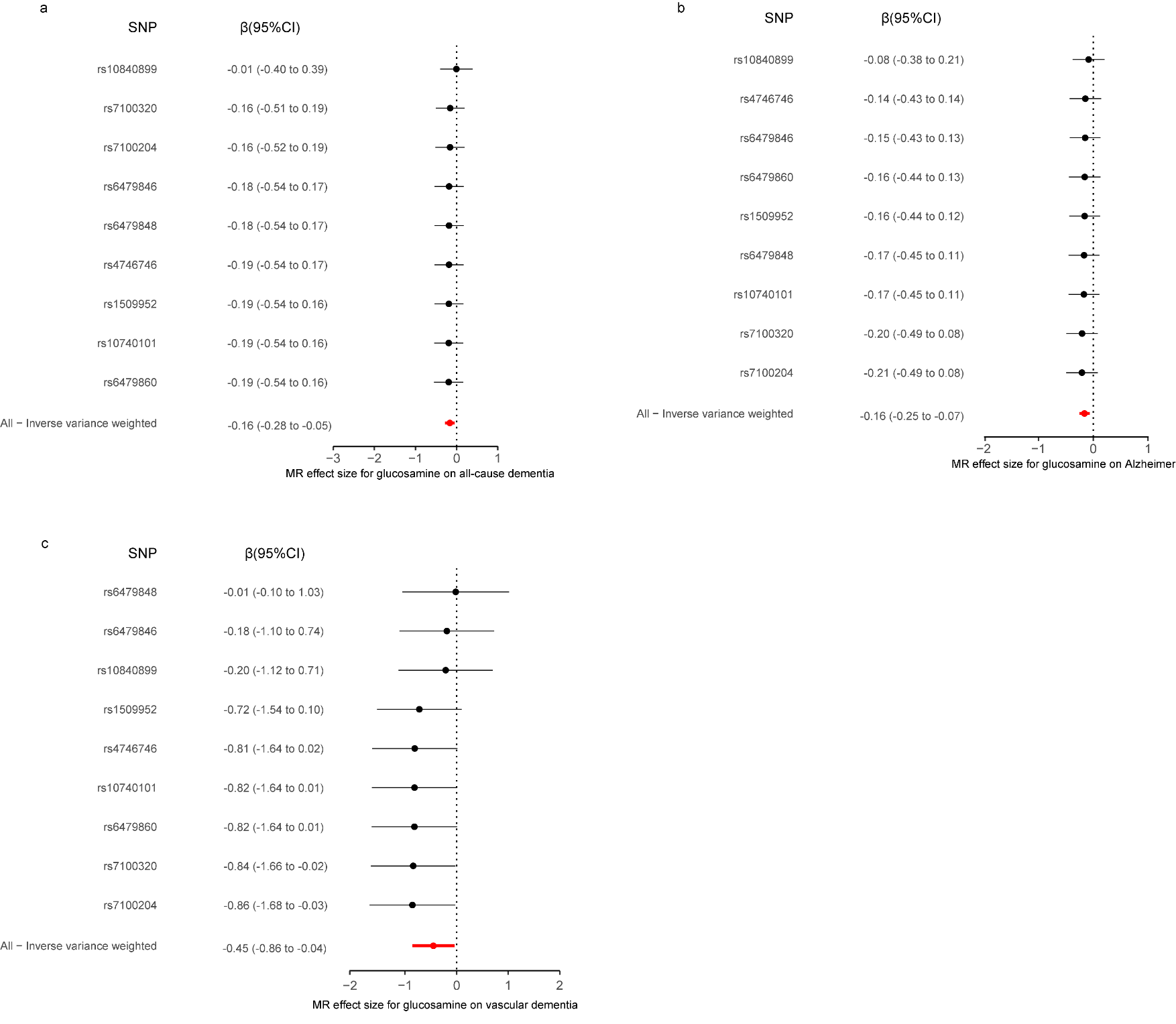
Figure S1. Forest plot for the relationship of regular glucosamine use with incident dementia.**

Forest plot of individual and combined single-nucleotide polymorphism (SNP) MR-estimated effects sizes for glucosamine supplement intake on (a) all-cause dementia, (b) Alzheimer’s disease and (C) vascular dementia. Error bars represent 95% confidence intervals.

**Figure S2. Leave-one-out analyses for SNPs associated with regular glucosamine use on incident dementia.**

**a b**


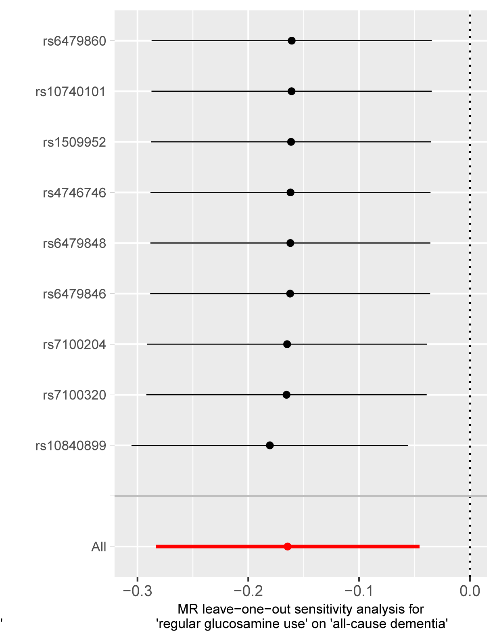

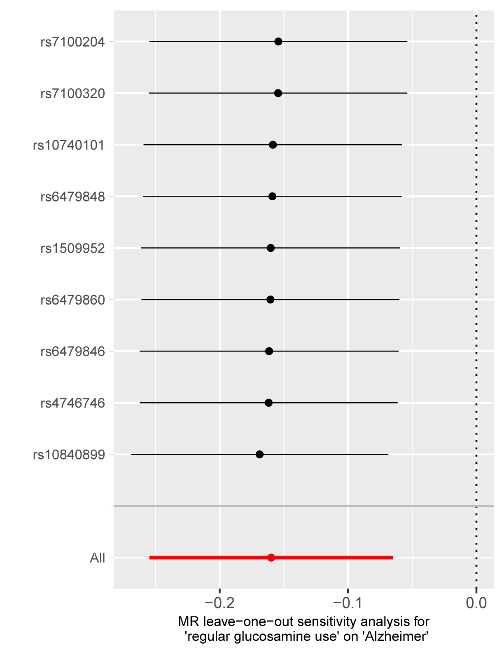


**c**


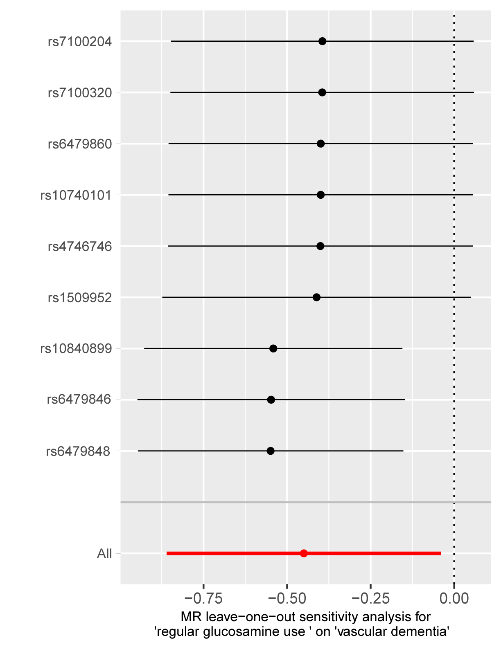


The leave-one-SNP out analysis was conducted to assess the influence of individual variants of regular glucosamine use (a) all-cause dementia, (b) Alzheimer’s disease and (c) vascular dementia. Error bars represent 95% confidence intervals.
